# Bryophyte Diversity in the Khaybar White Volcano Geopark (Saudi Arabia)—Floristic Patterns and Conservation Perspectives

**DOI:** 10.3390/plants14223423

**Published:** 2025-11-08

**Authors:** Vincent Hugonnot, Florine Pépin, Jan Freedman

**Affiliations:** 1 Independent Researcher, 43380 Blassac, France; flopepin@gmail.com; 2 Royal Commission for AlUla, 12512 Riyadh, Saudi Arabia; j.freedman@rcu.gov.sa

**Keywords:** Arabian Peninsula, bryophyte conservation, floristics, volcano, fumaroles, checklist

## Abstract

Recent bryological surveys conducted at the Khaybar White Volcano site (northwest Saudi Arabia) led to the documentation of 51 bryophyte species, including five liverworts and 46 mosses. Representing approximately 30% of the national bryophyte flora within less than 0.3% of the country’s surface, this site emerged as a regional hotspot of bryological diversity. A systematic catalog was compiled, presenting the biogeography, local distribution, demography, fertility, taxonomy and ecology of all recorded taxa. Notably, two Arabian endemics—*Crossidium deserti* and *Tortula mucronifera*—were identified in Khaybar, alongside six previously unknown on the Arabian Peninsula (*Anoectangium euchloron*, *Geheebia erosa*, *Grimmia capillata*, *Molendoa sendteriana*, *Pterygoneurum subsessile*, and *Ptychostomum torquescens*) and six species newly recorded for Saudi Arabia (*Anoectangium aestivum*, *Husnotiella revoluta*, *Syntrichia pagorum*, *Tortella nitida*, *Tortula lindbergii*, and *Tuerckheimia svihlae*). These findings highlighted the conservation value of Khaybar, whose unique geothermal microhabitats (active fumaroles) supported a suite of tropical and thermophilous species otherwise absent in northern Arabia, such as *Fissidens sciophyllus*, and *Plagiochasma eximium*. Comparative analysis with the AlUla region revealed a comparable species richness despite Khaybar’s smaller area and indicated substantial ecological divergence. While AlUla’s bryoflora was primarily associated with lithological heterogeneity, Khaybar’s was shaped by geothermal activity. Conservation recommendations emphasize the vulnerability of these specialized bryophyte communities to grazing, trampling, and climate change, and call for long-term monitoring, regulated access, and integration into national biodiversity management strategies.

## 1. Introduction

The Kingdom of Saudi Arabia (KSA), the largest country in Southwest Asia, spans approximately 2,150,000 km^2^—nearly three-quarters of the Arabian Peninsula [1]. Often perceived as uniformly arid, its landscapes are in fact highly diverse; vast rocky and sandy deserts dominate the central and eastern regions, while high mountain ranges, rising to 3700 m, stretch along the western escarpments near the Red Sea and toward the Yemeni border. The country’s biogeographical position at the crossroads of Africa, Asia, and Europe has profoundly shaped its flora and bryoflora. Although endemism is limited, the bryoflora encompasses six floristic elements [2], with a notable dominance of xerothermic species (Pangean and circum-Tethyan elements) and a consistent presence of tropical taxa with Southeast Asian–Arabian, African–Arabian, pantropical, or paleotropical affinities [3].

Bryophyte research in Saudi Arabia is relatively recent [4], but has grown steadily over the past decades. The first bryophyte checklist for the Arabian Peninsula, including Saudi Arabia, was published in 2000 [3], and later expanded to cover all of Southwest Asia in a comprehensive revision in 2020 [5]. The last national bryophyte checklist for Saudi Arabia was published by Hugonnot et al. [6]. Taking into account new species mentioned in [7,8], the bryophyte flora of Saudi Arabia now comprises 167 species: 136 mosses and 31 liverworts, with no hornworts recorded to date.

Volcanic regions of Saudi Arabia are likely to harbor a high diversity of bryophytes, although, to our knowledge, no study has been conducted specifically on this type of geological formation. The country has large, extensive volcanic fields (locally called harrats) across the western and northern areas, with a combined landmass of around 100,000 km^2^ [9]. The collision between the Arabian Peninsula and Europe, caused by the rifting of the Red Sea, led to large-scale mantle melting, resulting in several intercontinental volcanoes [9,10]. Due to the predominantly arid environment and lack of vegetation cover, the numerous volcanoes and extensive basalt lava flows are exposed, creating a range of different habitats. Bryophytes have been recorded as being very successful species on the hard lava flows in Hawaii [11,12,13] and Iceland [14,15]. Although Hawaii and Iceland have climates different from Saudi Arabia, the large diversity of bryophyte species recorded illustrates a significant potential for successful surveys in these habitats.

Harrat Khaybar, located in northwestern Saudi Arabia between AlUla and Medina, is one of the largest volcanic fields in the Kingdom. It covers approximately 14,000 km^2^ and is characterized by extensive basaltic lava fields, volcanic cones, and ephemeral watercourses. Volcanic substrates and residual geothermal activity create localized microclimates that mitigate aridity and favor the persistence of bryophytes, thereby making these volcanic habitats key refugia within an otherwise desert environment. Despite its geological and ecological uniqueness, the area had never been surveyed for bryophytes. During the winter of 2024 and 2025, targeted fieldwork was conducted across a 600 km^2^ area within this extensive region, particularly within protected zones and sites of ecological interest. This study provides the first bryophyte checklist for Khaybar, including a distribution atlas for each species, ecological notes, and conservation recommendations for this distinctive volcanic landscape.

## 2. Results

A total of fifty-one distinct taxa were observed, among which three (*Didymodon* cf. *rigidulus*, *Enthostodon* cf. *commutatus*, and *Fissidens* cf. *arnoldii*) are taxonomic uncertainties (Table 1). This total represents 30% of the total bryophytes recorded in Saudi Arabia.

Six species previously unknown to the Arabian Peninsula (*Anoectangium euchloron*, *Geheebia erosa*, *Molendoa sendtneriana*, *Pterygoneurum subsessile*, and *Ptychostomum torquescens*) and six species newly recorded for Saudi Arabia (*Anoectangium aestivum*, *Husnotiella revoluta*, *Syntrichia pagorum*, *Tortella nitida*, *Tortula lindbergii*, and *Tuerckheimia svihlae*) were recorded in Khaybar White Volcano Geopark.

With only five species being recorded, liverworts were very rare in the studied area, with the vast majority of bryophytes being true mosses (90.2% of the taxa). Four species belonged to the complex thalloid order Marchantiales, with only one liverwort from the order Fossombroniales. Although leafy liverworts have been recorded in southwestern Saudi Arabia, the overall taxonomic profile remained quite comparable to that of the country, with 81% of records in Khaybar White Volcano Geopark being mosses.

Pottiaceae were highly dominant in the bryophyte flora, as they are throughout Saudi Arabia and in AlUla County [6]. *Funariaceae* and *Bryaceae* were also relatively well represented in Khaybar White Volcano Geopark, AlUla County, and the country. Overall, the family representation was very similar to that of AlUla County, with only two notable differences: the presence of one species from the Brachytheciaceae and one from the Fossombroniaceae.

**Figure 1:**
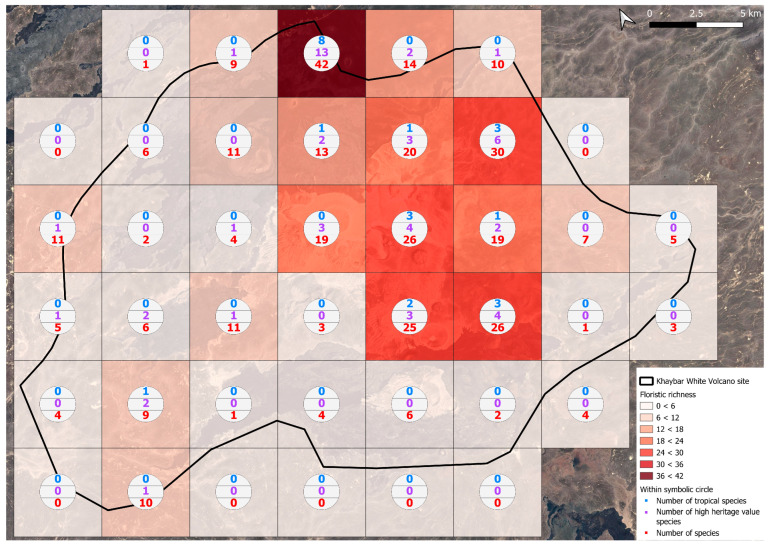
Floristic richness of Khaybar White Volcano Geopark.

The spatial distribution of bryophyte species richness across the Khaybar White Volcano Geopark is presented in Figure 1. Floristic richness per 5 × 5 km grid cell varied markedly, ranging from 0 to 42 species.

Species richness was highest in the northeastern quadrant of the study area, with one grid cell hosting 42 species. Several surrounding cells also showed elevated richness (≥30 species), particularly those situated in the central-eastern portion of the site. In contrast, many peripheral cells, especially along the southern and southwestern margins, displayed low richness (fewer than six species or no bryophytes recorded).

A spatial comparison of values within the symbolic circles revealed a positive correspondence between total bryophyte richness and the number of tropical and high heritage value species. Grid cells exhibiting the highest total species counts (≥30 species; dark red shading) consistently contained elevated numbers of both tropical taxa (up to 8) and high heritage value taxa (up to 13). This pattern was particularly evident in the northeastern and central-eastern parts of the Khaybar White Volcano Geopark. Conversely, cells with low overall species richness (white or light grey shading; <6 species) generally lacked tropical and heritage species, with most of these cells displaying zeros across both categories.

Tropical species were mainly recorded in the central and eastern parts of the site, with a maximum of 8 tropical taxa observed in a single grid cell. High heritage value species occurred in several grid cells, reaching a peak of 13 species in one northeastern cell.

### 2.1. Aloina rigida (Hedw.) Limpr. (Pottiaceae)

**Figure 2:**
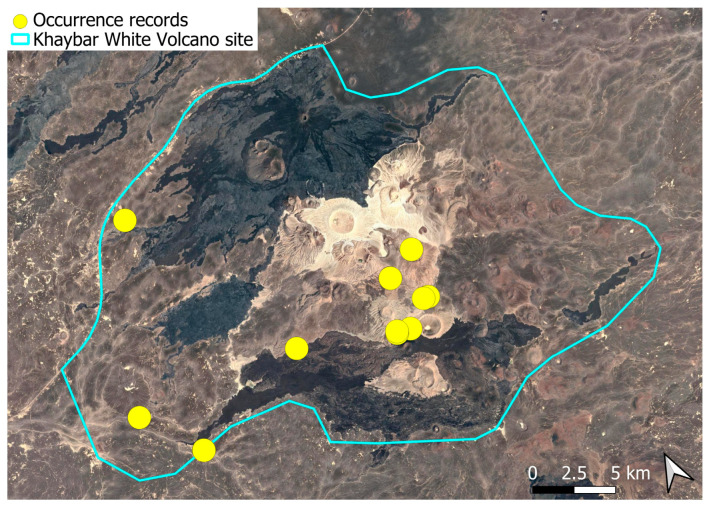
Distribution of *Aloina rigida* (Hedw.) Limpr. in Khaybar White Volcano Geopark.

A xerothermic Pangean species, widely distributed across the Arabian Peninsula, occurring in various arid and semi-arid habitats [3]. In the Khaybar White Volcano Geopark area, its distribution was sparse and discontinuous. Populations were few and spatially constrained (only 12 observations, see Figure 2). Nevertheless, fertile individuals were commonly observed.

Taxonomic determination remained challenging due to the difficulty in observing critical diagnostic characters, such as the differentiated hyaline leaf margin and the relatively thick stereid band. No noteworthy morphological variability was detected among the collected specimens.

Ecologically, this primarily terricolous species shows moderate specialization for dry, open habitats with minimal vegetation cover. This species was seen on older, eroded lava surfaces and was not recorded from the fresh basalt lavas. It preferentially colonized earthy banks, especially along wadi systems. Though occasionally observed on rock faces and volcanic slopes, these saxicolous occurrences appeared secondary and opportunistic.

### 2.2. Anoectangium aestivum (Hedw.) Spruce (Pottiaceae)

**Figure 3:**
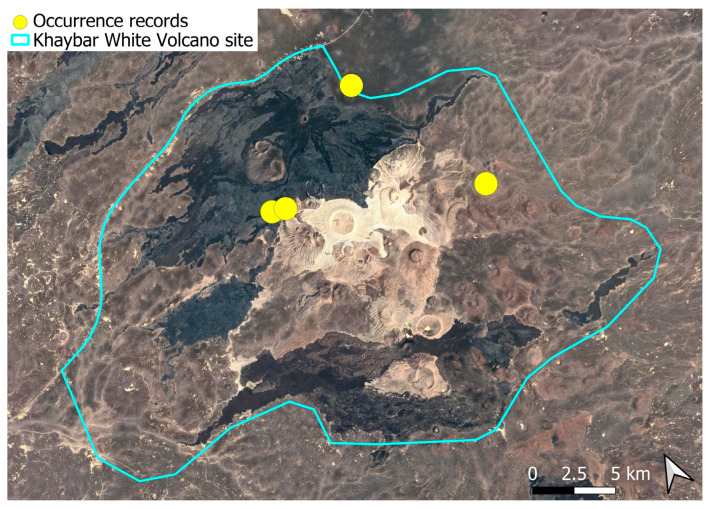
Distribution of *Anoectangium aestivum* (Hedw.) Spruce in and just outside the Khaybar White Volcano site.

A subcosmopolitan species with a distinct high montane character, known in the Arabian Peninsula only from a few records in southern Asir of Yemen, at around 2000 m elevation [16]. It was rare, found in the north-eastern part of Khaybar White Volcano Geopark and just outside the area (Figure 3). Local populations were consistently small and weakly developed, and always occurred in association with *A. euchloron*, which was typically much more abundant. No sexual organs or sporophytes were observed.

The species is strictly saxicolous, confined to unstable volcanic slopes in fumarolic zones.

### 2.3. Anoectangium euchloron (Schwägr.) Spruce (Pottiaceae)

**Figure 4:**
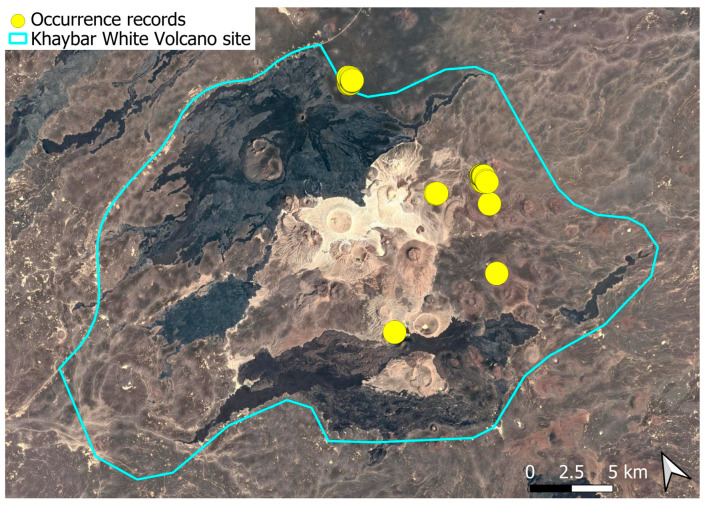
Distribution of *Anoectangium euchloron* (Schwägr.) Spruce in and just outside the Khaybar White Volcano Geopark area.

A pantropical species not previously recorded in the Arabian Peninsula, *A. euchloron* was found in the eastern half of the Khaybar White Volcano Geopark, exclusively on fumarolic volcanic slopes (Figure 4). While its distribution remained highly localized, it was frequent within its specialized microhabitat. Local populations were often well developed, sometimes covering several tens of cm^2^, and occasionally extending up to one square meter under favorable conditions. The species was entirely sterile, with no gametangia observed, like in *A. aestivum*.

Morphologically, *A. euchloron* is a highly distinctive species, readily identifiable in the field even within mixed stands with *A. aestivum*. Its consistently contorted leaf posture when dry, reminiscent of the genus *Barbula*, contrasts clearly with the feebly incurved, appressed leaves of *A. aestivum*. These characters show no observed intermediates, reinforcing its taxonomic validity despite Zander’s [17] proposed synonymy with *A. aestivum*. Based on field and herbarium observations from the Khaybar White Volcano Geopark area, we support the recognition of *A. euchloron* as a separate species, in agreement with the ecological and morphological distinctions highlighted by Arts & Sollman [18] in Ecuador. Although rhizoidal tubers were reported as a distinguishing trait in that study, we did not observe them in our material despite targeted investigation.

Ecologically, the species is strictly saxicolous, exclusively restricted to thermally influenced volcanic habitats (fumaroles).

### 2.4. Bryum dichotomum Hedw. (Bryaceae)

**Figure 5:**
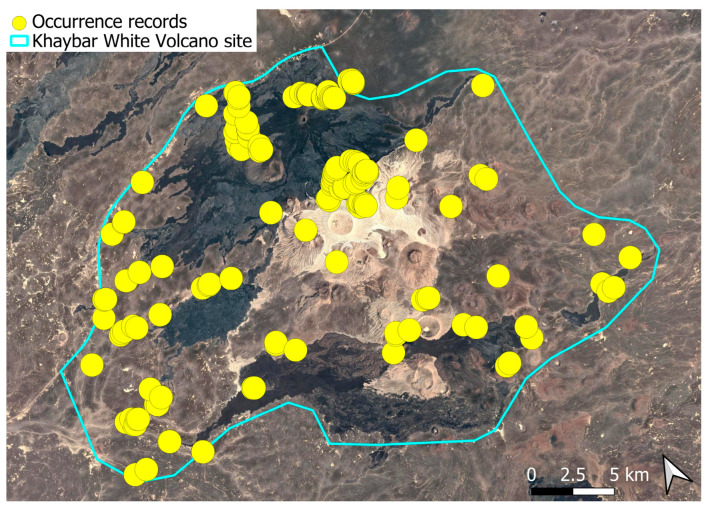
Distribution of *Bryum dichotomum* Hedw. In and just outside the Khaybar White Volcano Geopark area.

This cosmopolitan species is widely distributed across the Arabian Peninsula and is also abundant throughout the Khaybar White Volcano Geopark (Figure 5). It is by far the most frequently recorded bryophyte in the area. Although most populations were restricted to a few square centimeters, more extensive colonies—sometimes spanning several square meters—were recorded, particularly in lava flows where conditions appeared especially favorable. No gametangia or sporophytes were observed in the region, but vegetative propagation appears to be the dominant mode of reproduction, as similarly noted at the AlUla site [6].

A broad taxonomic framework was applied here for *Bryum dichotomum*, following the interpretation proposed in [19].

The species demonstrates considerable ecological plasticity and is primarily terricolous, typically occurring in earthy substrates, temporary grasslands, and flood-prone zones. It is also commonly found in semi-protected niches such as animal burrow entrances, beneath lava slabs, or near cave mouths.

### 2.5. Crossidium aberrans Holz. & E. B. Bartram (Pottiaceae)

**Figure 6:**
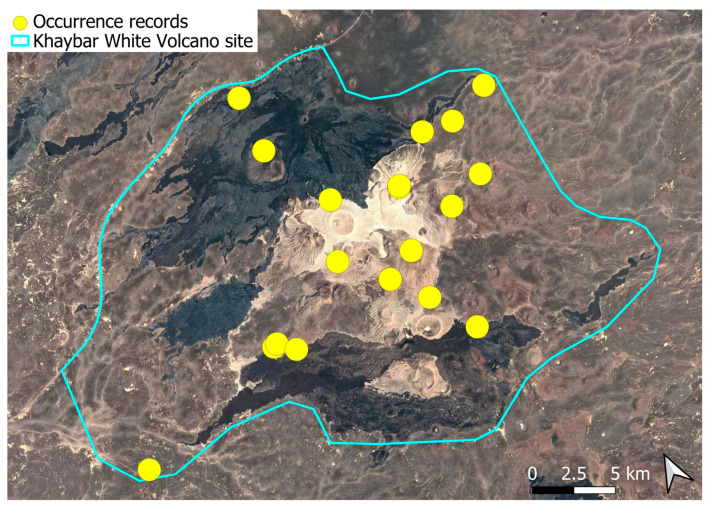
Distribution of *Crossidium aberrans* Holz. & E. B. Bartram in Khaybar White Volcano Geopark.

A circum-Tethyan species, widespread and often locally abundant across the Arabian Peninsula [3]. In the Khaybar White Volcano Geopark, it was moderately frequent (Figure 6). Populations were generally well developed and comparatively extensive relative to other species sharing similar ecological niches. Fertile individuals were only occasionally observed.

Taxonomic resolution within the genus *Crossidium* remains incomplete, and specific populations in the region pose identification challenges, as discussed in Hugonnot et al. [6]. Despite these uncertainties, the populations observed in Khaybar exhibited consistent traits compatible with the species concept adopted here.

Ecologically, the species is primarily terricolous, favoring dry, mineral-rich soils. It typically colonizes earthy microhabitats and sheltered soil accumulations beneath boulders, but is also found in partially exposed sites such as screes and lava flows.

### 2.6. Crossidium crassinervium (De Not.) Jur. (Pottiaceae)

**Figure 7:**
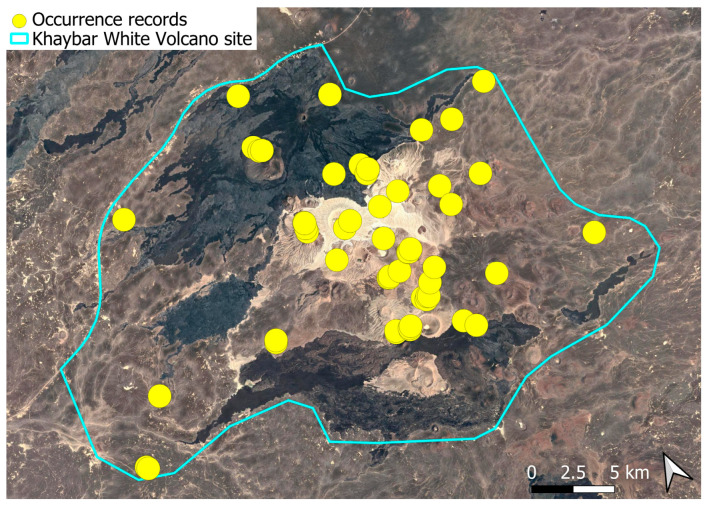
Distribution of *Crossidium crassinervium* (De Not.) Jur. in Khaybar White Volcano Geopark.

This circum-Tethyan species is broadly distributed and frequently locally abundant across the Arabian Peninsula [3]. In the Khaybar White Volcano Geopark, it was frequent in suitable microhabitats (Figure 7). Populations were generally well developed and often more extensive than those of co-occurring species. Fertile specimens were only occasionally encountered.

Primarily terricolous, this species exhibits notable ecological flexibility, colonizing a range of dry, open habitats. It is frequently found on soil accumulations beneath boulders or along banks, but also establishes on rocky substrates, including screes, lava flows, and outcrops.

### 2.7. Crossidium deserti W. Frey & Kürschner (Pottiaceae)

**Figure 8:**
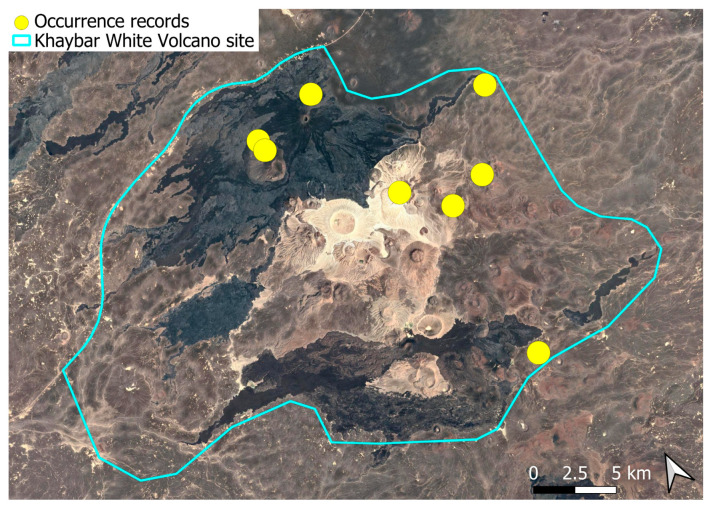
Distribution of *Crossidium deserti* W. Frey & Kürschner in Khaybar White Volcano Geopark.

A widely distributed circum-Tethyan element, frequently exhibiting local abundance in the Arabian Peninsula [3]. In the Khaybar White Volcano Geopark, it was localized and rare mainly in the northern part of the area (Figure 8), and generally formed relatively small populations, especially in comparison to many other congeners. Fertile individuals were only occasionally observed.

Despite the number of studies devoted to the genus *Crossidium* [20,21,22], its taxonomy remains uncertain and would benefit from a re-evaluation using molecular approaches. The status of *C. deserti* in particular remains ambiguous and warrants further investigation.

Ecologically, the species is specialized for dry, fully exposed habitats. It is predominantly terricolous, growing in loose, mineral-rich soils and in earthy pockets beneath stones, but also occurs in fissures and on fractured volcanic rock surfaces.

### 2.8. Crossidium squamiferum (Viv.) Jur. (Pottiaceae)

**Figure 9:**
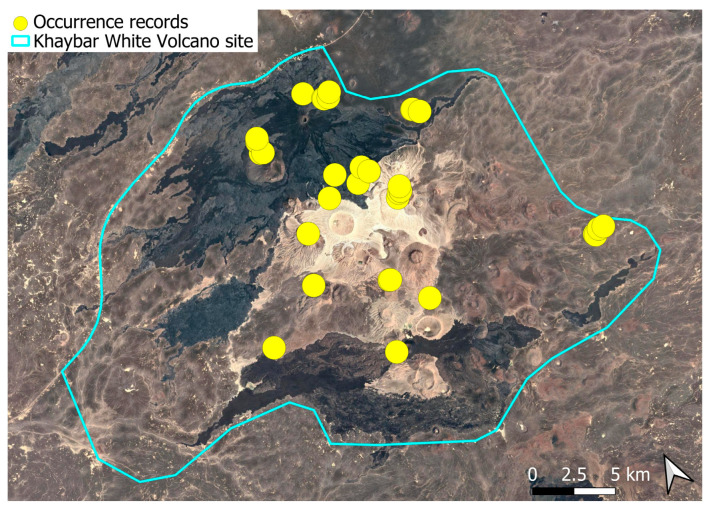
Distribution of *Crossidium squamiferum* (Viv.) Jur. in Khaybar White Volcano Geopark.

A circum-Tethyan species, widespread and often locally abundant across the Arabian Peninsula [3]. In the Khaybar White Volcano Geopark (Figure 9), this species was frequent and was one of the most vigorous and productive, forming substantial cushions and establishing larger populations than most other taxa occupying similar habitats. Sporophytes were occasionally produced, but typically in small numbers.

Ecologically, this species displays a mixed substrate preference. It can be found on both basalt lava and comendite volcanoes, thriving in rocky environments—such as fissures, outcrops, and block faces—and in earthy microsites, particularly in soil pockets protected beneath stones.

### 2.9. Didymodon cf rigidulus Hedw. (Pottiaceae)

**Figure 10:**
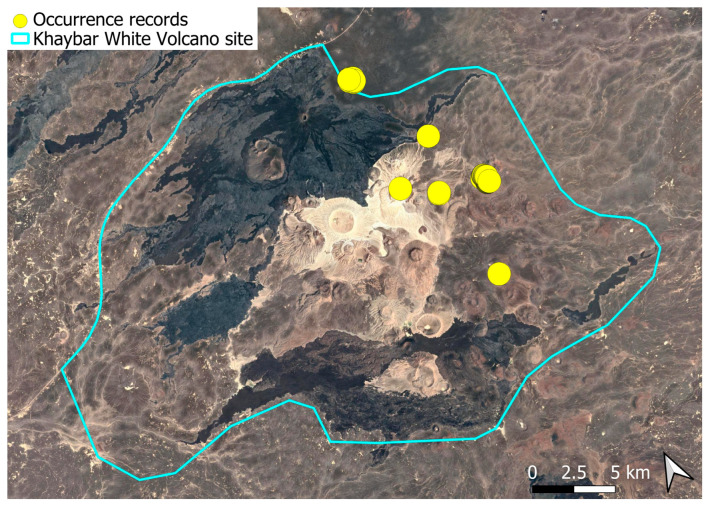
Distribution of *Didymodon cf rigidulus* Hedw. In and just outside Khaybar White Volcano Geopark.

A xerothermic Pangean species, rare but seemingly widespread across the Arabian Peninsula [3]. In and just outside Khaybar White Volcano Geopark, it was relatively frequent but of restricted occurrence, mainly in the northeastern part of the site (Figure 10). Populations were often locally abundant, forming extensive mats on suitable substrates. While always sterile, the presence of leaf axil gemmae indicated vegetative reproduction as the dominant strategy.

Morphologically, the material observed in this area showed several deviations from the typical concept of *Didymodon rigidulus*. Notably, plants displayed a characteristic habit with spreading basal leaves that become erect and acuminate toward the apex. The leaves were predominantly unistratose, occasionally bistratose at the tip, and the basal laminal cells were short and nearly isodiametric. These traits raised questions about the taxonomic circumscription of the species and suggested that further study was needed to clarify its identity.

Ecologically, the species is strictly saxicolous, confined to thermally influenced or highly mineral substrates, including looser material on volcanic slopes, and barren outcrops.

### 2.10. Didymodon desertorum (J. Froehl.) J. A. Jiménez & M. J. Cano (Pottiaceae)

**Figure 11:**
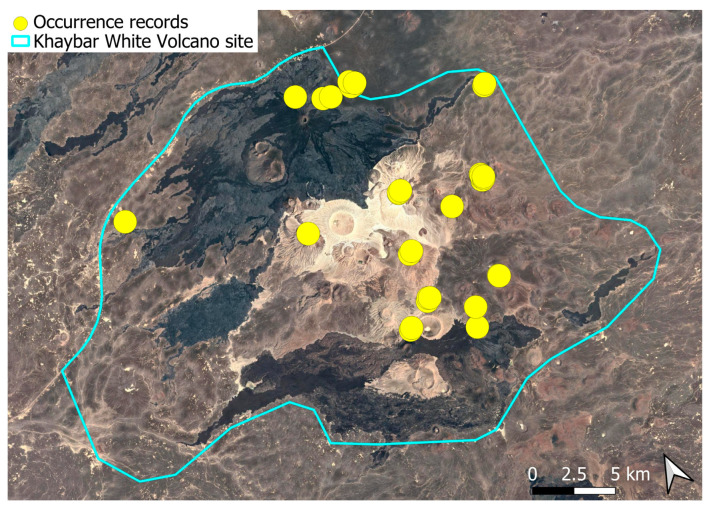
Distribution of *Didymodon desertorum* (J. Froehl.) J. A. Jiménez & M. J. Cano in and just outside Khaybar White Volcano Geopark.

This circum-Tethyan taxon is relatively widespread across the Arabian Peninsula [3] and was frequent in and just outside Khaybar White Volcano Geopark (Figure 11). It was often inconspicuous within its populations, typically represented by only a few scattered stems. Although archegonia were occasionally observed, sporophytes have never been recorded, and populations consist exclusively of sterile individuals.

Ecologically, the species exhibits considerable versatility, occupying a broad range of dry microhabitats on various geological substrates. While predominantly saxicolous—common on rock faces, exposed slopes, and fissures—it is also found in more terricolous contexts, such as earthy banks and soil pockets beneath stones, especially within wadi systems.

### 2.11. Entosthodon cf commutatus Durieu & Mont. (Funariaceae)

**Figure 12:**
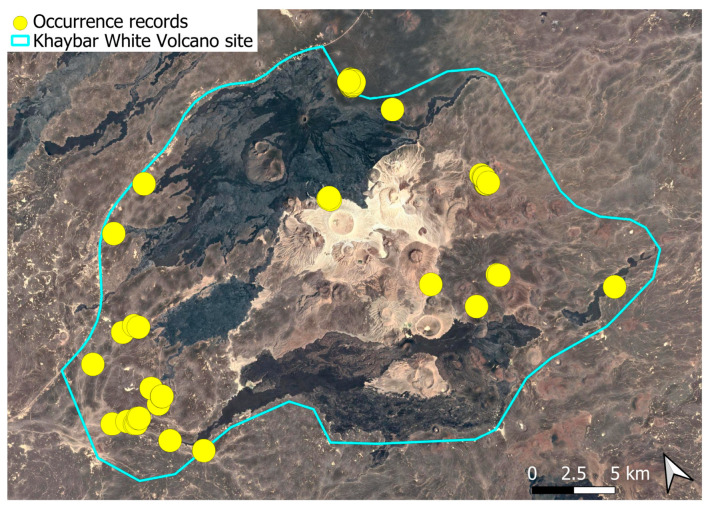
Distribution of *Entosthodon* cf. *commutatus* Durieu & Mont. in and just outside Khaybar White Volcano Geopark.

A circum-Tethyan, Mediterranean species, apparently widespread across the Arabian Peninsula based on unpublished records. *Entosthodon* cf. *commutatus* was previously discussed in Hugonnot et al. [6] and was very frequently encountered in and just outside Khaybar White Volcano Geopark, where it seemed to avoid the central part of fresh basalt and was rarely found on the comendite volcanoes (Figure 12). Populations were often extensive, and unlike observations from AlUla [6], sporophytes were frequently produced, often abundantly so, especially in more humid microsites.

It is likely often confused with *Entosthodon attenuatus* (Dicks.) Bryhn, a species considered relatively common in Saudi Arabia, but not observed during our surveys. In contrast, *E.* cf. *commutatus* was regularly found and identified based on its diagnostic features: a long but non-excurrent nerve, pale brown rhizoids (never cherry red), and a capsule with a broad mouth when dry, and a well-developed peristome.

Ecologically, the species is mainly terricolous, occupying loose to compacted soils in dynamic, periodically disturbed habitats such as wadis, flood-prone lava fields, and earthy slopes. It also occasionally colonizes fissured rocks and fumarolic zones.

### 2.12. Enthostodon muhlenbergii (Turner) Fife (Funariaceae)

**Figure 13:**
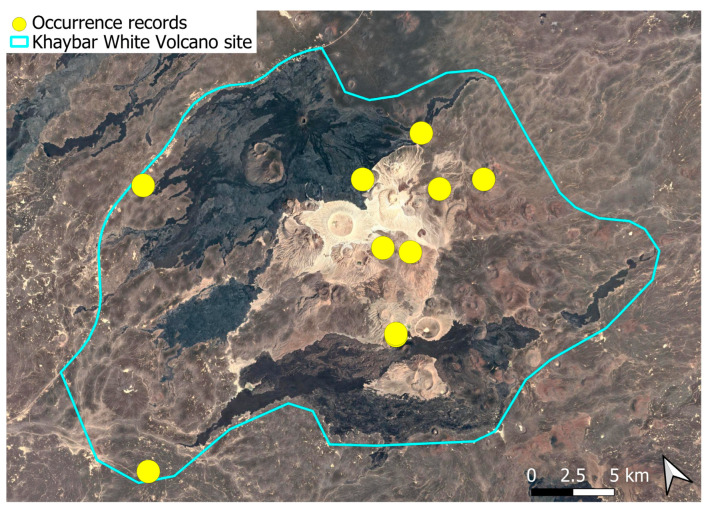
Distribution of *Entosthodon muhlenbergii* (Turner) Fife in Khaybar White Volcano Geopark.

A circum-Tethyan species moderately distributed across the Arabian Peninsula, but relatively rare [3]. It was rarely observed, mainly in the north-eastern and central parts of the Khaybar White Volcano Geopark, with isolated occurrences in the west (Figure 13). It was typically encountered in low densities and was mostly sterile, its sporophytes being very rarely formed.

Ecologically, the species is primarily terricolous, favoring protected soil pockets beneath stones and within wadi systems. It is also occasionally found in rocky canyons and on older, eroded lava fields, but does not appear to establish on the fresher basalt lava flows.

### 2.13. Fissidens cf. arnoldii R. Ruthe (Fissidentaceae)

**Figure 14:**
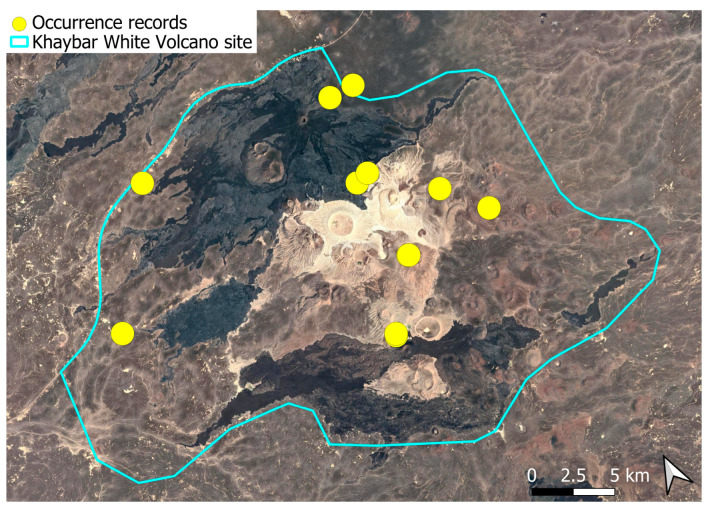
Distribution of *Fissidens* cf. *arnoldii* R. Ruthe in and just outside Khaybar White Volcano Geopark.

A circum-Tethyan species, *Fissidens* cf. *arnoldii* is broadly distributed across the Arabian Peninsula, including hyper-arid regions [3]. In contrast, it was moderately frequent within and just to the north of the Khaybar White Volcano Geopark (Figure 14), where it occurred only sporadically and in low abundance. The sporophytes were very rare.

Firmly established in the regional flora since the publication of [23], this species exhibits morphological differences from typical European *Fissidens arnoldii*, as previously noted [6] and confirmed during our own observations. The presence of morphologically similar taxa in parts of Asia further supports the need for a thorough taxonomic reassessment. Field populations were often sterile and morphologically reduced, complicating identification.

Ecologically, the species is distinctly terricolous, with a strong affinity for protected or humidified microsites. It typically grows on fine soils within wadis, along earthy banks, and beneath boulders, though it may occasionally occur in rock fissures, shaded canyons, and fumaroles.

### 2.14. Fissidens sciophyllus Mitt. (Fissidentaceae)

**Figure 15:**
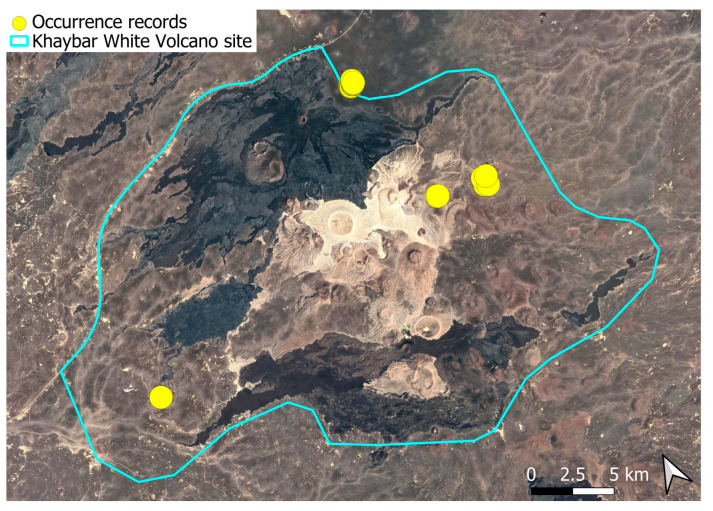
Distribution of *Fissidens sciophyllus* Mitt. in and just outside Khaybar White Volcano Geopark.

A paleotropical species, with its northernmost population in southwestern Arabia just below 20° latitude [3], making the occurrence in and just outside the Khaybar White Volcano Geopark a highly isolated outpost. It was moderately frequent and restricted to fumarolic zones (Figure 15). Populations were minute, consisting of a few, poorly developed, and consistently sterile individuals—no gametangia or sporophytes were observed.

The taxon is currently placed within *sciophyllus* Mitt., a morphologically heterogeneous group. The “*arabicus*-expression”, initially described as an independent species [24], is characterized by relatively short costae. However, costal length is known to vary broadly within the species complex, which clearly requires taxonomic revision.

Ecologically, this species is highly specialized, strictly limited to saxicolous microhabitats under geothermal influence.

### 2.15. Fissidens crispus Mont. (Fissidentaceae)

**Figure 16:**
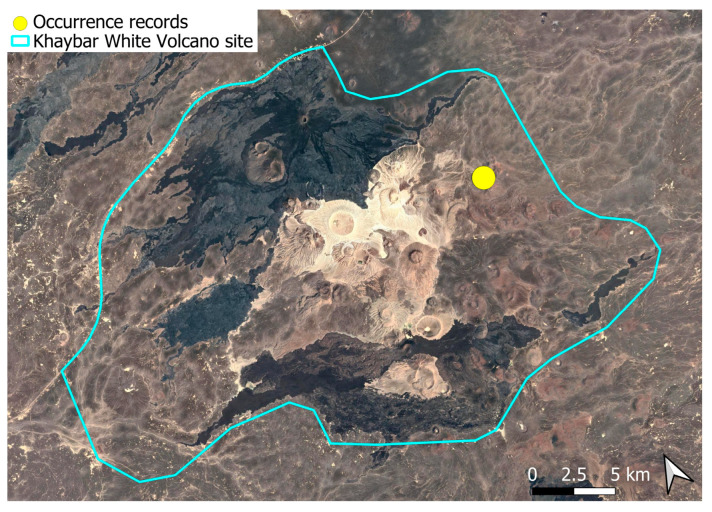
Distribution of *Fissidens crispus* Mont. in Khaybar Geopark.

A circum-Tethyan species, scattered and rare across the Arabian Peninsula. At the Khaybar White Volcano site, it was scarce and confined exclusively to fumarolic zones (Figure 16). The population detected was of minimal extent and comprised very few individuals. Sporophytes were rare, but all observed sporophyte-bearing specimens were unequivocally assigned to this species.

Ecologically, the species is strictly saxicolous, restricted to geothermal volcanic slopes and fumaroles.

### 2.16. Fossombronia caespitiformis (Raddi) De Not. ex Rabenh. subsp. caespitiformis (Fossombroniaceae)

**Figure 17:**
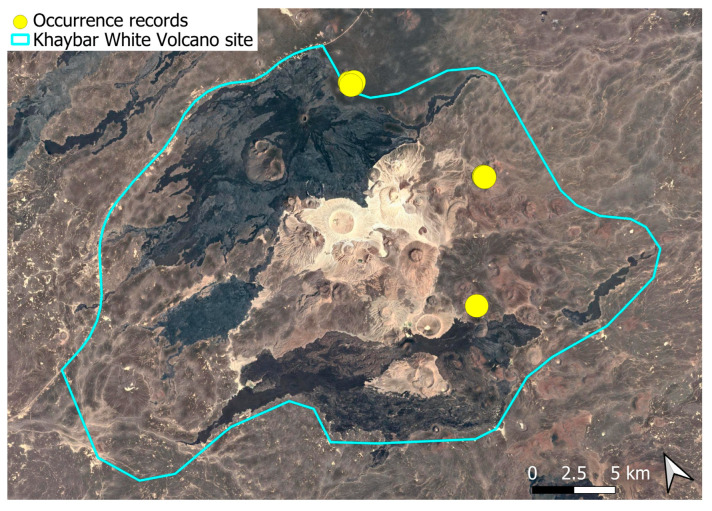
Distribution of *Fossombronia caespitiformis* (Raddi) De Not. *ex Rabenh. subsp. caespitiformis* in and just outside Khaybar White Volcano Geopark.

A circum-Tethyan liverwort, scattered and rare across the Arabian Peninsula, primarily along the Red Sea corridor [3]. In and just to the north of the Khaybar White Volcano Geopark, it was moderately frequent, and confined exclusively to fumarolic zones (Figure 17). Populations were tiny, often limited to just a few shoots, and appeared to persist at the edge of edaphic moisture tolerance. Fertile individuals were rare.

All observed spore-bearing specimens were unequivocally assigned to this species. Additionally, vegetative characters proved stable across all collections. Notably, this is the only non-Marchantioid liverwort recorded in the area.

Ecologically, the species is strictly saxicolous, restricted to geothermal volcanic slopes and fumaroles.

### 2.17. Funaria hygrometrica Hedw. (Funariaceae)

**Figure 18:**
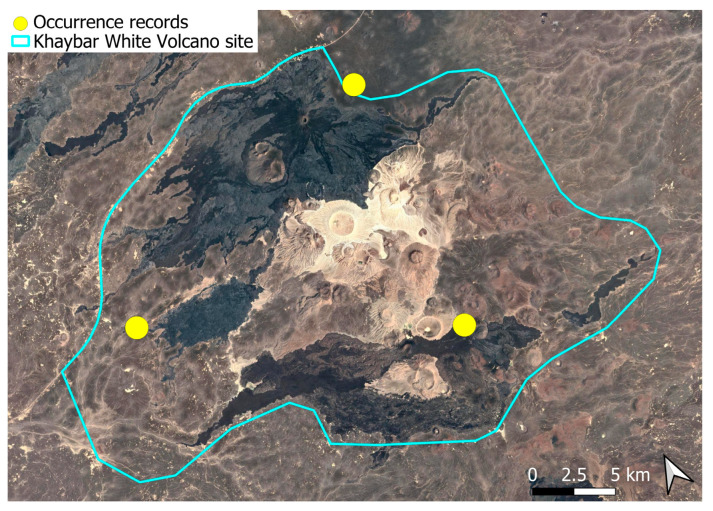
Distribution of *Funaria hygrometrica* Hedw. in and just outside Khaybar White Volcano Geopark.

A sub-cosmopolitan species widely distributed in southwestern Asia, *Funaria hygrometrica* was previously known to the Arabian Peninsula only through a record in Kuwait [5], until its recent discovery in Saudi Arabia, where it had evidently been neglected [6]. It remained scarce at the local scale (Figure 18). Despite its limited occurrence, populations were often well developed and freely fertile, with regular production of sporophytes.

Ecologically, this species is clearly terricolous, favouring disturbed, nutrient-rich soils in environments subject to transient moisture. It typically colonizes earthy patches in grasslands, entrances of animal burrows, and flood-prone zones.

### 2.18. Geheebia erosa (J.A. Jiménez & J. Guerra) J.A. Jiménez & M.J. Cano (Pottiaceae)

**Figure 19:**
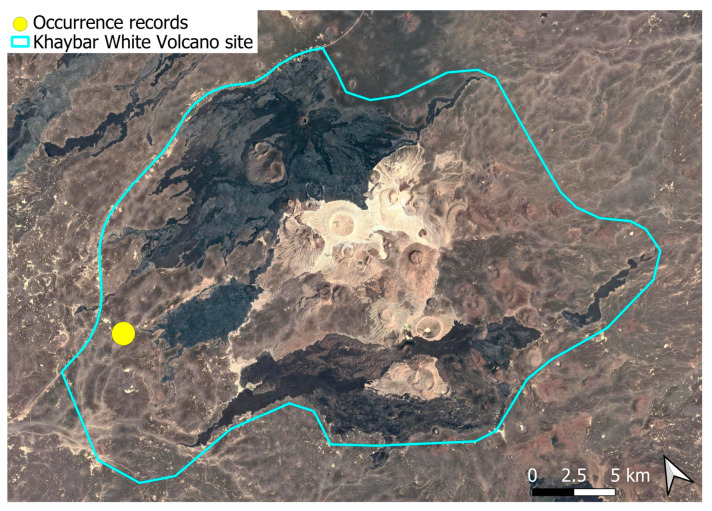
Distribution of *Geheebia erosa* (J.A. Jiménez & J. Guerra) J.A. Jiménez & M.J. Cano in Khaybar White Volcano Geopark.

A xerothermic Pangean species not previously recorded in southwestern Asia. In the Khaybar White Volcano Geopark, it was scarce as it was found in a single, spatially restricted locality (Figure 19), with minimal population size and number of individuals. All observed specimens were completely sterile, though the presence of rhizoidal tubers was confirmed.

*Geheebia* is a recently segregated genus from the broad and morphologically diverse *Didymodon* complex, based on both morphological characters and multilocus phylogenetic analyses [25]. The delimitation of the *Didymodon tophaceus* complex has also been revised [26], highlighting substantial morphological overlap and ITS-based non-monophyly. Accordingly, *G. siccula* and *G. erosa* have been proposed as subspecies of *G. tophacea*. However, following the framework of [5], we provisionally maintain these taxa as separate species due to their consistent morphological distinctiveness.

Ecologically, this moss is strictly terricolous, restricted to earthy banks at wadi margins. It shows a clear preference for mineral soils periodically affected by surface runoff and seasonal moisture input.

### 2.19. Grimmia capillata De Not. (Grimmiaceae)

**Figure 20:**
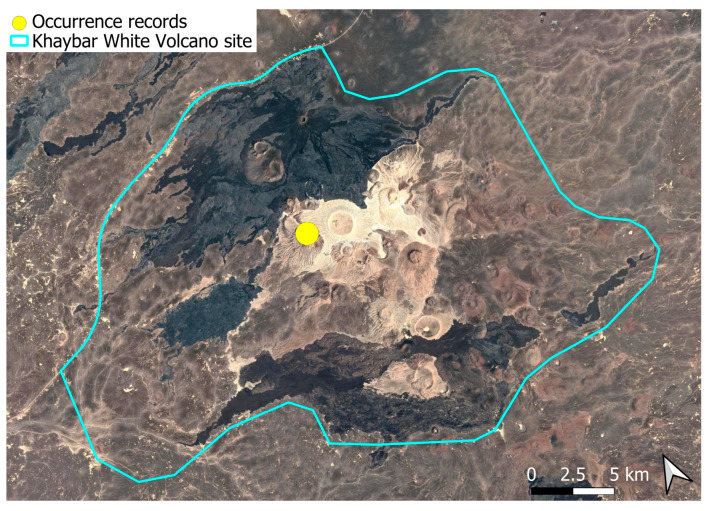
Distribution of *Grimmia capillata* De Not. in Khaybar White Volcano Geopark.

A circum-Tethyan taxon previously unrecorded on the Arabian Peninsula, even when *G. mesopotamica* Schiffn. is considered a synonym, an option rejected by Kürschner & Frey [5]. *Grimmia capillata* is known from a single population in the Khaybar White Volcano Geopark, where it was obviously scarce (Figure 20). Although not abundant overall, it formed several well-structured cushions within its restricted locality. The species was fully fertile, with well-developed sporophytes.

*G. capillata* is not generally considered morphologically close to *G. orbicularis*, yet distinguishing the two can be challenging in the field in the local context, primarily because of the unusual, very short seta of the latter. In the regional area, *G. orbicularis* lamina is consistently pluristratose, with uniformly pilose leaves and no clear differentiation between proximal and perichaetial leaves [6]. In contrast, *G. capillata* is characterized by a catenulate arrangement of reduced, epilose proximal leaves, sharply transitioning to pilose, slightly membranous perichaetial leaves. According to Maier [27], these upper leaves may sometimes appear less hyaline. The capsule morphology also aids in separation: in *G. capillata*, the annulus persists even in mature capsules, and the peristome teeth are broadly triangular with at most three divisions. In *G. orbicularis*, the annulus is deciduous, the teeth are more rectangular, typically divided into four parts, and often show anastomosing patterns.

Ecologically, this species is strictly saxicolous, restricted to volcanic slopes and bare rocky outcrops. It demonstrates a strong affinity for hard, exposed substrates in arid environments, likely benefiting from low competition and intense solar radiation.

### 2.20. Grimmia orbicularis Bruch ex Wilson (Grimmiaceae)

**Figure 21:**
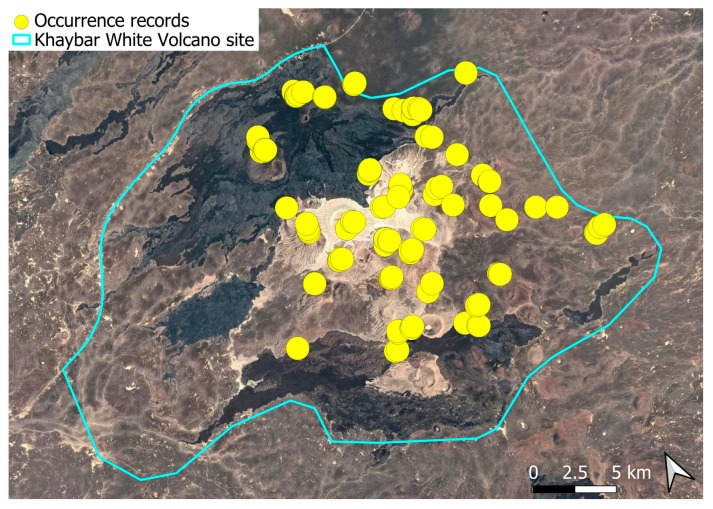
Distribution of *Grimmia orbicularis* Bruch ex Wilson in and just outside Khaybar White Volcano Geopark.

A northern species with an apparent Mediterranean affinity, *Grimmia orbicularis* was far more frequent and widespread than related taxa in and just outside the Khaybar White Volcano Geopark (Figure 21). Its populations were generally well developed and often locally abundant. Sporophytes were frequently present, though rarely produced in large numbers.

Our observations confirm the broad morphological variability previously documented in AlUla County [6], encompassing differences in leaf structure and pigmentation within and between populations.

Ecologically, this species is a typical saxicolous moss, thriving across a wide range of rocky microhabitats. It is commonly found on outcrops, block faces, fissures, cliff systems, and consolidated pozzolanic deposits. It also occurs in transitional microsites, such as earthy zones beneath stones and within wadis.

### 2.21. Gymnostomum calcareum Nees & Hornsch. var. calcareum (Pottiaceae)

**Figure 22:**
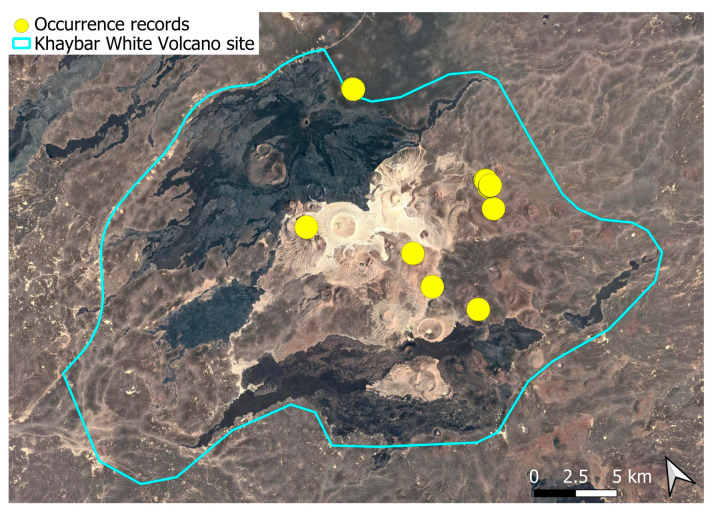
Distribution of *Gymnostomum calcareum* Nees & Hornsch. in and just outside Khaybar White Volcano Geopark.

*Gymnostomum calcareum* is a cosmopolitan species, relatively widespread across the Arabian Peninsula [3], though only moderately frequent in and just outside the Khaybar White Volcano Geopark (Figure 22). At this site, it was observed in limited populations. Compared to *G. mosis*, which can form expansive mats over several square meters in favourable conditions, *G. calcareum* was less abundant and formed more restricted colonies. A single fertile occurrence (showing a colliculate exothecium) was recorded; gemmae were not observed.

Its long, ribbon-shaped, or ligulate leaves distinguish it morphologically within the local bryoflora.

Ecologically, this species is saxicolous, confined to volcanic slopes, fumarolic zones, and rocky habitats within wadis. None of the *Gymnostomum* species observed in the Geopark is associated with tufa-forming communities, which are virtually absent here.

### 2.22. Gymnostomum mosis (Lorentz) Jur. & Milde (Pottiaceae)

**Figure 23:**
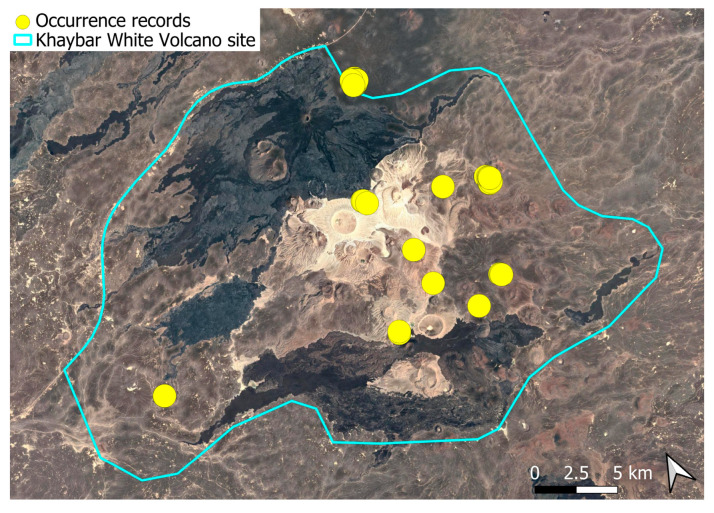
Distribution of *Gymnostomum mosis* (Lorentz) Jur. & Milde in and just outside Khaybar White Volcano Geopark.

*Gymnostomum mosis* is a circum-Tethyan species, relatively widespread across the Arabian Peninsula [3] and particularly well represented in Khaybar White Volcano Geopark, and just outside to the north (Figure 23), where it was considerably more frequent and abundant than *G. calcareum*. Its sporophytes were rare.

Fertile specimens of *G. mosis* display a cylindrical capsule with a colliculate exothecium, a distinct red rim composed of transversely elongated cells, and a long, rostrate operculum. Sterile individuals are characterized by the absence of gemmae, relatively short leaves, and irregular marginal stratosity, with some leaves exhibiting mostly unistratose margins. Leaf shape is somewhat variable; apices are typically rounded.

Ecologically, *G. mosis* is primarily saxicolous, growing on volcanic slopes, rocky outcrops, and mineral soils in wadis and fumarolic areas.

### 2.23. Husnotiella revoluta Cardot

**Figure 24:**
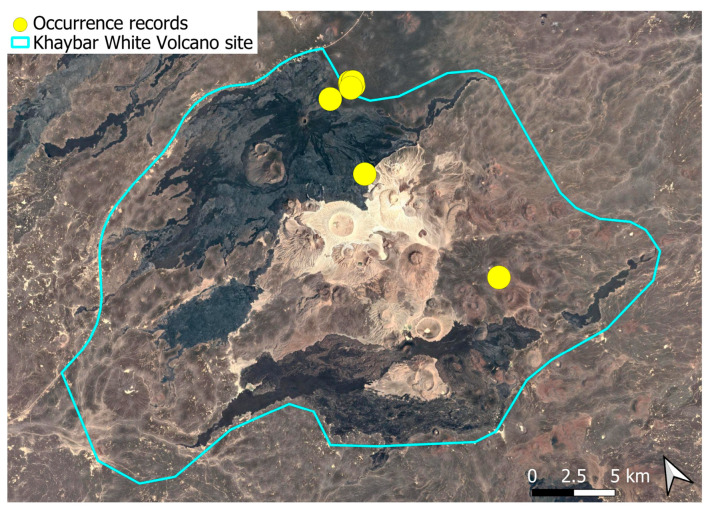
Distribution of *Husnotiella revoluta* Cardot in and just outside Khaybar White Volcano Geopark.

A pantropical species, *Husnotiella revoluta* is extremely rare on the Arabian Peninsula, with a single record in Yemen, near San‘a’ (Wadi Dahr, 2200 m) [28]. The population found in and just outside the Khaybar White Volcano Geopark area (Figure 24) represented a highly disjunct occurrence, isolated both geographically and ecologically. The species was very scarce locally. All observed material was completely sterile, with no sporophytes present.

Local populations exhibited morphological variability well beyond what was documented by Jiménez et al. [28], confirming instead the broader polymorphism historically associated with this taxon. The leaves were oblong, rounded at the apex, with a distally dilated costa that extended laterally into the lamina, penetrating the foliar tissue—traits consistent with classical descriptions of the species.

Ecologically, this moss is strictly saxicolous, growing on volcanic slopes near fumarolic zones.

### 2.24. Hymenostylium hildebrandtii (Müll. Hal.) R. H. Zander (Pottiaceae)

**Figure 25:**
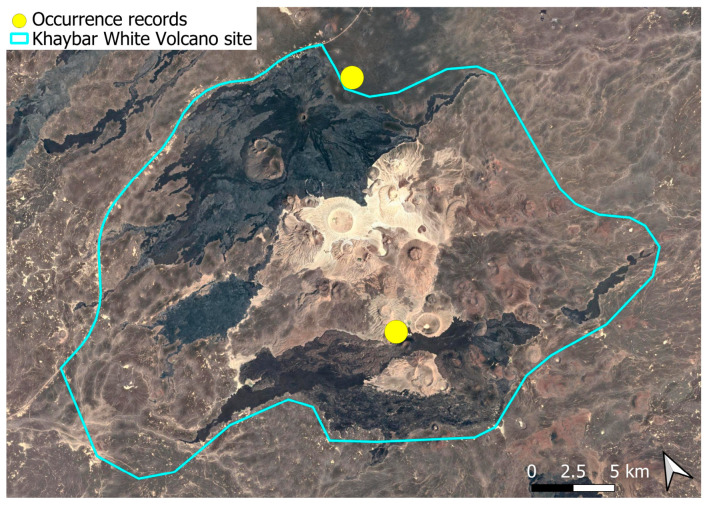
Distribution of *Hymenostylium hildebrandtii* (Müll. Hal.) R. H. Zander in and just outside Khaybar White Volcano Geopark.

This Paleotropical species is rare across the southwestern Arabian Peninsula [29] and has been recorded in Makkah Province [30], but was very rare within the Khaybar White Volcano Geopark and just to the north of the area (Figure 25). It occurred in small, isolated populations. Sporophytes were not observed in the Khaybar region, although they are known from more southern populations. All local individuals are sterile.

Ecologically, the species is highly localized, favoring transiently moist microhabitats in erosion-prone wadi beds and fumaroles.

### 2.25. Microbryum davallianum (Sm.) R.H. Zander (Pottiaceae)

**Figure 26:**
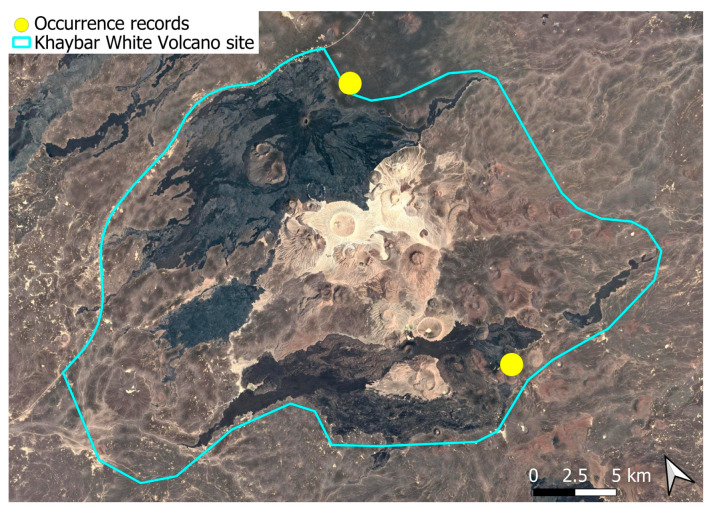
Distribution of *Microbryum davallianum* (Sm.) R.H. Zander in and just outside Khaybar White Volcano Geopark.

A circum-Tethyan species considered relatively frequent across the Arabian Peninsula [3], though typically under-recorded due to taxonomic difficulties associated with the genus. In and just to the north outside the Khaybar White Volcano Geopark area, it was scarce and of isolated occurrence (Figure 26). It was also surprisingly much less abundant than the other local species of the same genus. Sporophytes were commonly produced, but their diagnostic value was limited. Frequent developmental abortion prevented reliable spore observation, resulting in a substantial amount of undetermined or uncertain material across collections.

*Microbryum* species are classic pioneers, quickly colonizing bare, compacted substrates where competition is minimal. This species is no exception, being strictly terro-saxicolous and confined to geothermal habitats such as fumaroles and unstable volcanic slopes.

### 2.26. Microbryum starckeanum (Hedw.) R.H. Zander (Pottiaceae)

**Figure 27:**
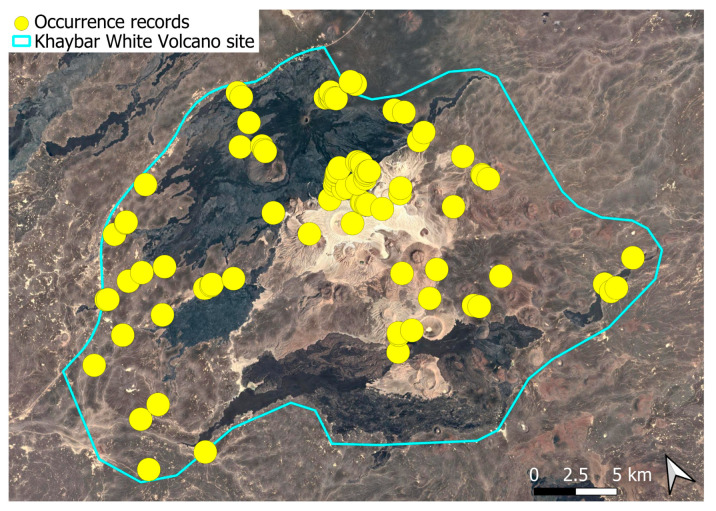
Distribution of *Microbryum starckeanum* (Hedw.) R.H. Zander in and just outside Khaybar White Volcano Geopark.

*Microbryum starckeanum* is a cosmopolitan species considered relatively frequent across the Arabian Peninsula [3] and was among the most commonly encountered bryophytes in and just outside the Khaybar White Volcano Geopark (Figure 27). Its frequent occurrence and often high abundance made it one of the characteristic components of the local bryoflora. The sporophytes were frequently produced, but they often aborted (see *M. davallianum*).

Reflecting its ecological flexibility, *M. starckeanum* is observed across a range of contrasting habitats. It is predominantly terricolous, favouring earthy banks, flood-prone soils, and sheltered pockets beneath stones. However, it also readily colonizes transitional and partially saxicolous environments such as screes, lava flows, and rock fissures.

### 2.27. Molendoa sendtneriana (Bruch & Schimp.) Limpr. (Pottiaceae)

**Figure 28:**
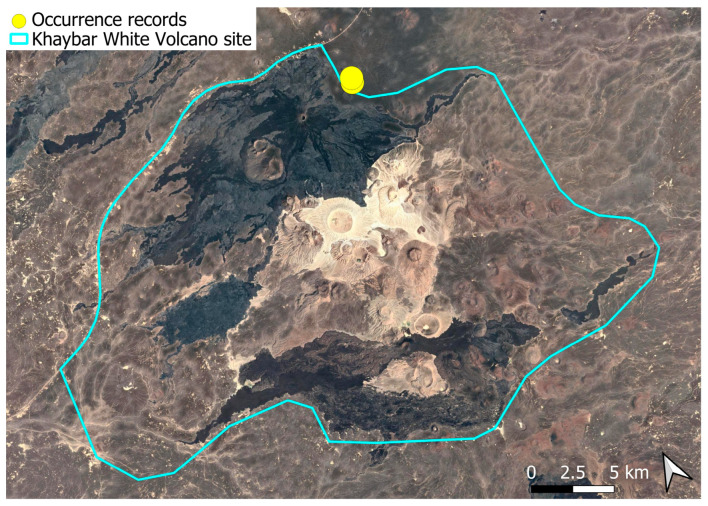
Distribution of *Molendoa sendtneriana* (Bruch & Schimp.) Limpr. just outside Khaybar White Volcano Geopark.

This species corresponds to a pantropical chorological element. It was scarce in the Khaybar White Volcano Geopark. Although it has not been previously reported in Saudi Arabia or the Arabian Peninsula, it is, in fact, frequent (pers. obs.) in the Asir mountain range, where it occurs in rocky and often wooded wadis. Its apparent absence from regional floristic inventories likely results from previous oversight. It is also known from a broad intercontinental range, including Europe, Northeast and Eastern Asia, Tropical Africa, North, Central, and South America [31]. The species was found in a single micro-population, consisting of a few poorly developed individuals just outside Khaybar White Volcano Geopark (Figure 28). No sporophytes were observed; all individuals were sterile.

Misidentifications are likely due to the difficulty of observing key taxonomic characters, especially the presence of highly reduced lateral branches bearing female gametangia [31]. Anatomically, the costa contains two stereid bands [32].

Ecologically, the species is strictly saxicolous, with its occurrences restricted to fumarolic environments.

### 2.28. Plagiochasma eximium (Schiffn. ex Steph.) Steph. (Aytoniaceae)

**Figure 29:**
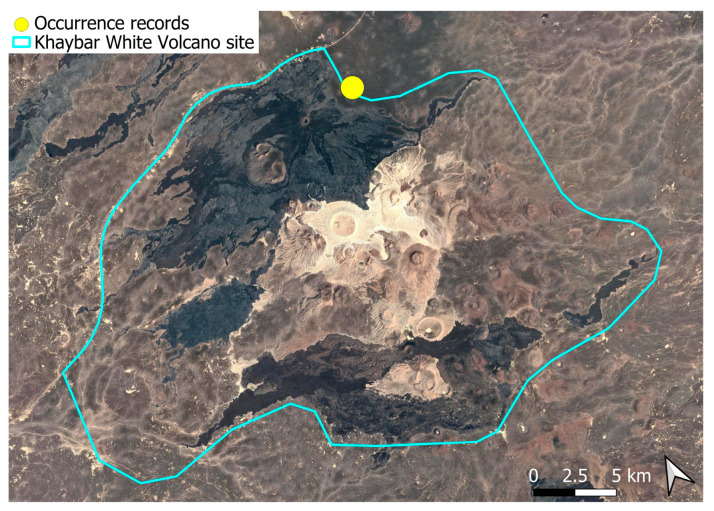
Distribution of *Plagiochasma eximium* (Schiffn. ex Steph.) Steph. just outside the Khaybar White Volcano Geopark.

A Palaeotropical taxon with a tropical African distribution, this species is known from the mountainous Asir region in southwestern Arabia, with its northernmost historical record near the latitude of Makkah [3]. The population just outside the Khaybar White Volcano Geopark represents the northernmost occurrence of the species within its Afro–Southwest Asian range. Outside Ethiopia, where it is more widespread [33], the species remains rare. It was scarce locally (Figure 29). The species was fertile locally.

Its taxonomy is challenging, and it may be confused with *P. beccarianum* Steph. However in the Khaybar material, it was distinguished by a thallus exceeding 6 mm in width, narrowly oblong-lanceolate appendages on the ventral scales, and elaters that were either spiral-less or possess interrupted spirals. This contrasts with *P. beccarianum*, which has a smaller thallus (less than 5 mm), triangular, attenuate scales, and consistently spiraled elaters [33].

Ecologically, this liverwort is highly specialized and strictly saxicolous, restricted to fumarolic zones. The species has also been previously reported elsewhere from volcanic caves [33].

### 2.29. Plagiochasma rupestre (J. R. Forst. & G. Forst.) Steph. (Aytoniaceae)

**Figure 30:**
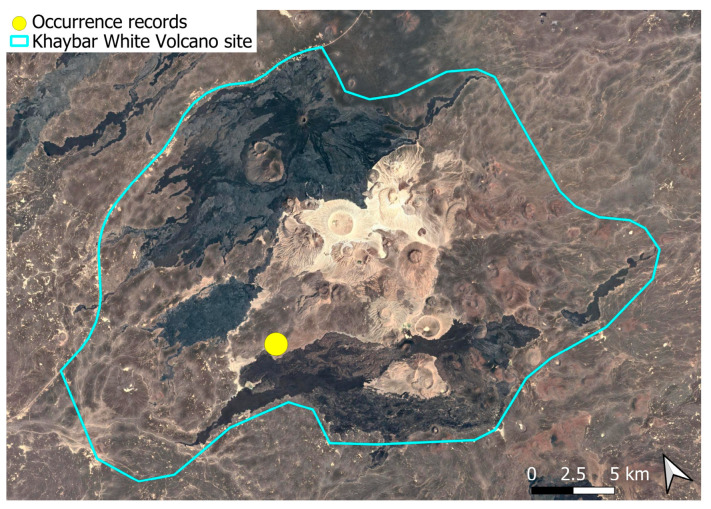
Distribution of *Plagiochasma rupestre* (J. R. Forst. & G. Forst.) Steph. in Khaybar White Volcano Geopark.

This xerothermic Pangean taxon is one of the most frequent and abundant marchantioid liverworts across the Arabian Peninsula. However, its presence in the Khaybar White Volcano Geopark was surprisingly very rare (Figure 30), mirroring observations in AlUla County [6]. This scarcity contrasted with its regional ubiquity and raises questions about local ecological constraints [6]. In the Khaybar White Volcano Geopark, *Plagiochasma rupestre* was found in minimal numbers, with only a few thalli observed in rock fissures. Some individuals were fertile, bearing carpocephala.

Ecologically, the species is saxicolous, favoring shaded or semi-exposed rocky microsites on cliff ledges and soil-filled crevices beneath boulders.

### 2.30. Pterygoneurum subsessile (Brid.) Jur. (Pottiaceae)

**Figure 31:**
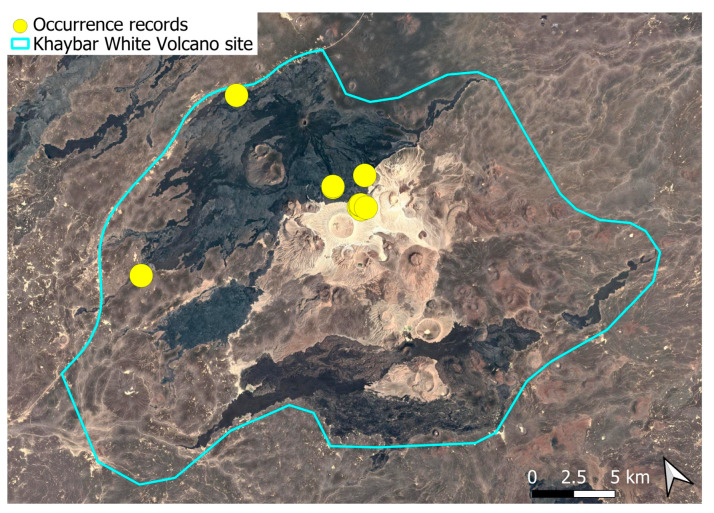
Distribution of *Pterygoneurum subsessile* (Brid.) Jur. in Khaybar White Volcano Geopark.

A xerothermic Pangean species with a distinctly northern character, previously known only in Israel and Turkey [5], and newly recorded for the Arabian Peninsula. In the Khaybar White Volcano Geopark, this rare species was observed in a few localized populations (Figure 31). Nonetheless, some populations were well established and could cover several square meters, sometimes forming nearly pure stands. Despite being fully fertile, sporophyte development was often abortive, limiting effective sexual reproduction.

Ecologically, the species is strictly terricolous, confined to bare or sparsely vegetated soil patches, particularly within young lava fields.

### 2.31. Ptychostomum pseudotriquetrum (Hedw.) J.R.Spence & H.P.Ramsay (Bryaceae)

**Figure 32:**
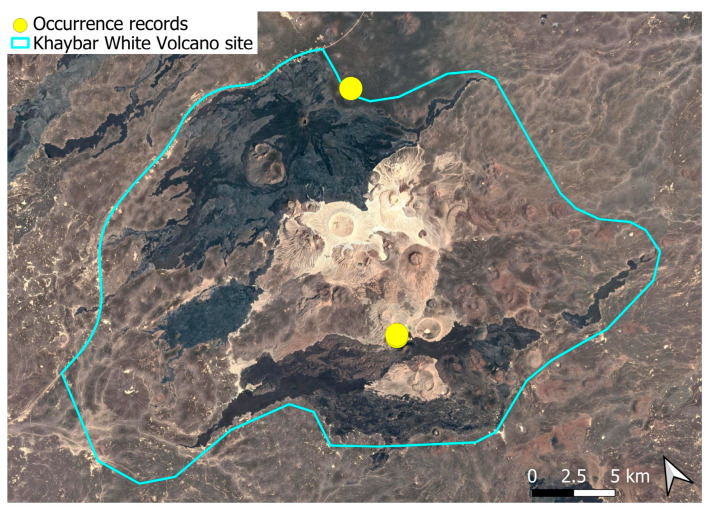
Distribution of *Ptychostomum pseudotriquetrum* (Hedw.) J. R. Spence & H. P. Ramsay in and just outside Khaybar White Volcano Geopark.

A sub-cosmopolitan species, recently discovered on the Arabian Peninsula in southwest Yemen [34] and more recently in AlUla County, where it remains uncommon [6]. In and just outside the Khaybar White Volcano Geopark, it was similarly rare (Figure 32), with populations limited to a small number of diminutive individuals. All observed material was sterile.

Ecologically, this moss is predominantly saxicolous, growing on consolidated volcanic substrates, lava flows, and rocky canyon walls. It also occurs in transitional microsites such as soil-filled crevices beneath boulders and within scree deposits.

### 2.32. Ptychostomum torquescens (Bruch & Schimp.) Ros & Mazimpaka

**Figure 33:**
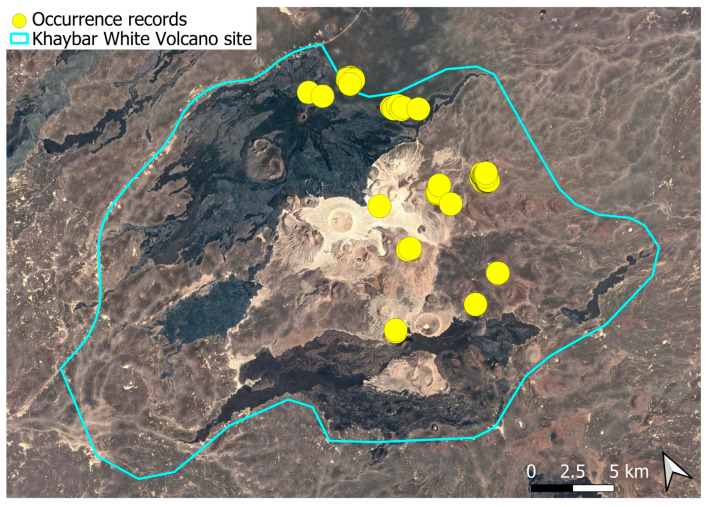
Distribution of *Ptychostomum torquescens* (Bruch & Schimp.) Ros & Mazimpaka in and just outside Khaybar White Volcano Geopark.

A subcosmopolitan species not previously recorded in the Arabian Peninsula [19]. In and just outside the Khaybar White Volcano Geopark, it was common and even locally abundant, particularly in fumarolic zones (Figure 33), where it formed well-developed colonies composed of robust, fully fertile individuals. Gametangia were frequently observed, while sporophytes were rare.

The species is known to be synoicous, though this condition is not consistently expressed. Many colonies exhibit a mixture of unisexual buds—potentially leading to confusion with the dioicous *P. capillare*—and synoicous inflorescences, highlighting some variability in reproductive expression. This was previously underlined by Syed [35].

Ecologically, the species is strictly saxicolous and strongly associated with geothermal habitats. It is confined to fumaroles and volcanic slopes.

### 2.33. Riccia cavernosa Hoffm. (Ricciaceae)

**Figure 34:**
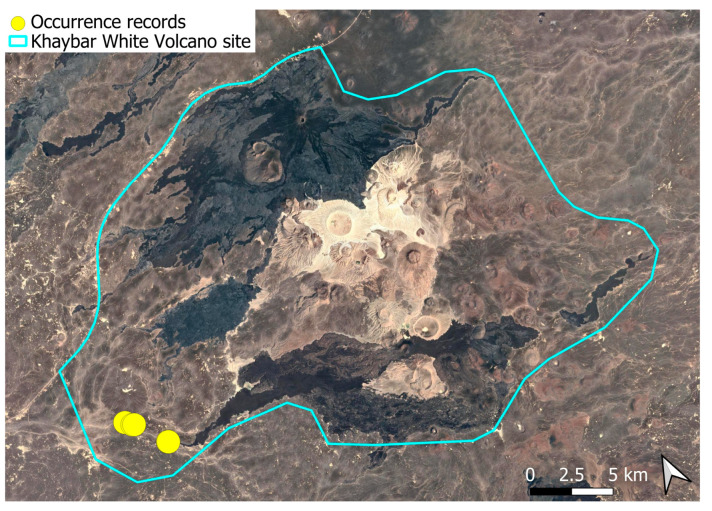
Distribution of *Riccia cavernosa* Hoffm. in Khaybar White Volcano Geopark.

This cosmopolitan species, of xerothermic Pangean origin, is widely distributed across the Arabian Peninsula, with a notable concentration of records along the Red Sea coast [3]. In the Khaybar White Volcano Geopark, however, it was moderately frequent due to the scarcity of suitable habitats, which were only present in the southwestern part (Figure 34). The populations were small (several tenth of thalli). Remarkably, the species almost always produced sporophytes, even when individuals were highly reduced and measured only a few millimeters in size.

As previously noted in AlUla County [6], the taxonomic identity of the local populations warrants further investigation, as subtle morphological variation suggests possible regional differentiation.

Ecologically, this liverwort is strictly terricolous, confined to transient soil habitats such as sandy wadi banks and exposed mudflats.

### 2.34. Scorpiurium circinatum (Brid.) M. Fleisch. & Loeske (Brachytheciaceae)

**Figure 35:**
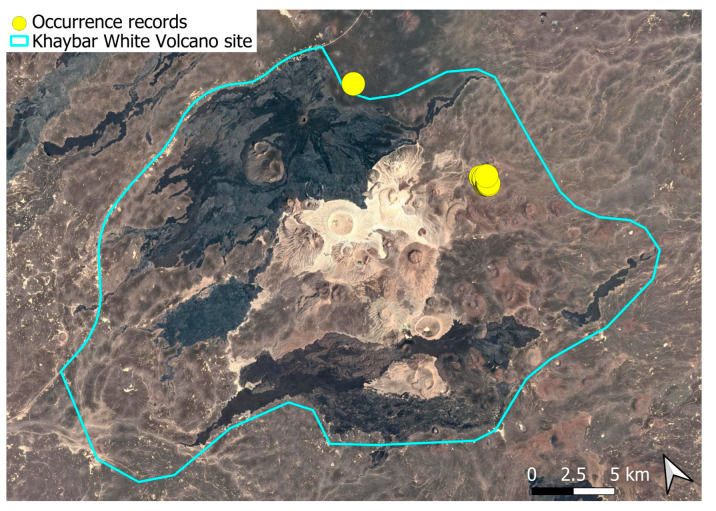
Distribution of *Scorpiurium circinatum* (Brid.) M. Fleisch. & Loeske in and just outside Khaybar White Volcano Geopark.

A circum-Tethyan (Mesogean) taxon, *Scorpiurium circinatum* is strictly confined to the southwestern Arabian Peninsula. In and just outside the Khaybar White Volcano site, it was frequent (Figure 35), and represented a notable biogeographical outlier, being the only pleurocarpous moss recorded north of the latitude of Makkah—excluding the Mediterranean *Fabronia pusilla* [3]. It was typically found in very small quantities, forming inconspicuous patches. The species was entirely sterile, with no sporophytes observed, and was frequently highly reduced vegetatively.

Ecologically, it is strictly saxicolous and closely linked to geothermal substrates, occurring exclusively on volcanic slopes in fumarolic zones.

### 2.35. Syntrichia caninervis Mitt. var. caninervis (Pottiaceae)

**Figure 36:**
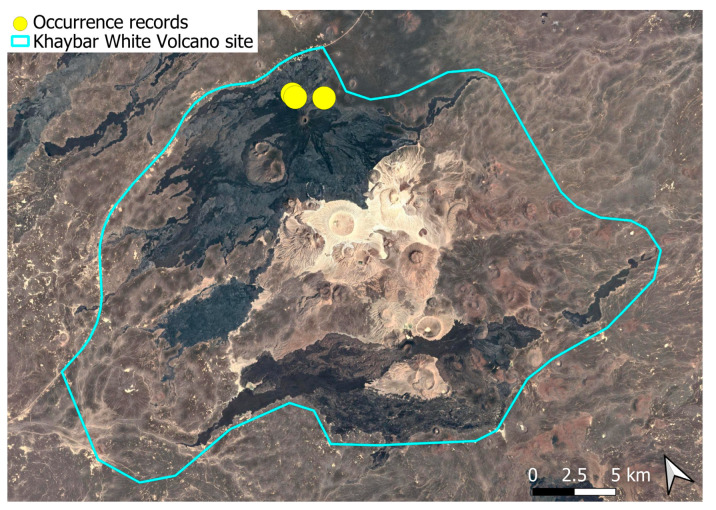
Distribution of *Syntrichia caninervis* Mitt. var. *caninervis* in Khaybar White Volcano Geopark.

The genus *Syntrichia* comprises mainly xerophytic species adapted to arid and semi-arid climates. Alongside *Tortula*, this genus is the most species-rich in the Khaybar White Volcano Geopark, with five species represented. *Syntrichia caninervis* var. *caninervis* is a xerothermic Pangean taxon, widely distributed across the Arabian Peninsula [3]. In the Khaybar region (Figure 36), it was rare, and occurred in the form of diminutive individuals, forming tiny and scattered colonies. It was entirely sterile there.

Ecologically, this moss is primarily terricolous, typically colonizing bare mineral soil within lava fields and nearby earthy microsites.

### 2.36. Syntrichia fragilis (Taylor) Ochyra (Pottiaceae)

**Figure 37:**
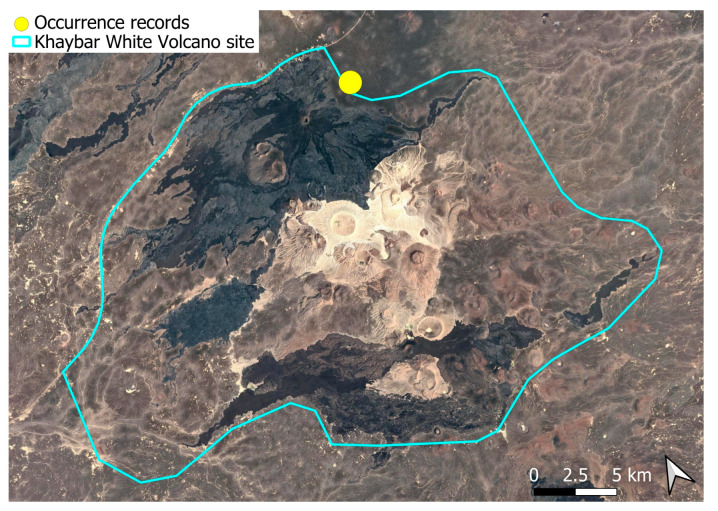
Distribution of *Syntrichia fragilis* (Taylor) Ochyra just outside the Khaybar White Volcano Geopark.

A pantropical species with a relictual Tertiary xerotropical affinity, *Syntrichia fragilis* is known from the Asir Mountains in Yemen and Saudi Arabia, but had not previously been recorded north of the latitude of Makkah [3]. Just outside the boundary to the Khaybar White Volcano Geopark, it was very rare, being represented by a single colony (Figure 37) observed in a fumarolic zone. It occurred in very low quantities and in a highly reduced vegetative state. The species was entirely sterile.

Ecologically, *S. fragilis* is strictly saxicolous, confined to exposed, mineral-rich rock surfaces on volcanic slopes, within fumaroles.

### 2.37. Syntrichia laevipila Brid. (Pottiaceae)

**Figure 38:**
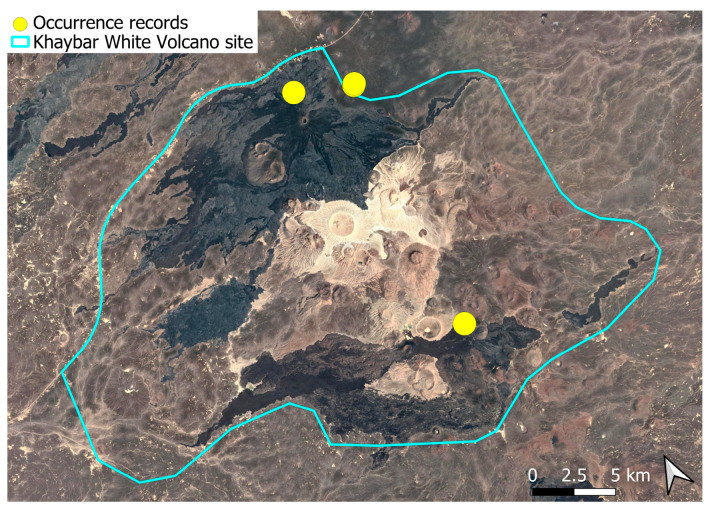
Distribution of *Syntrichia laevipila* Brid. in and just outside the Khaybar White Volcano Geopark.

A circum-Tethyan species confined to the southwestern Arabian Peninsula, where it is rare and primarily corticolous, growing on *Juniperus excelsa* and *Acacia origena* in escarpment woodlands [20]. It was rare in and just outside the Khaybar White Volcano Geopark (Figure 38). Plants were vegetatively reduced, forming very small, inconspicuous colonies that are difficult to detect in the field. It was locally entirely sterile, with no sporophytes observed.

Although principally corticolous outside Khaybar, this species is also moderately saxicolous here, occurring on volcanic slopes, lava flows, and rocky outcrops, and occasionally extending into nearby earthy microsites.

### 2.38. Syntrichia pagorum (Milde) J.J. Amann (Pottiaceae)

**Figure 39:**
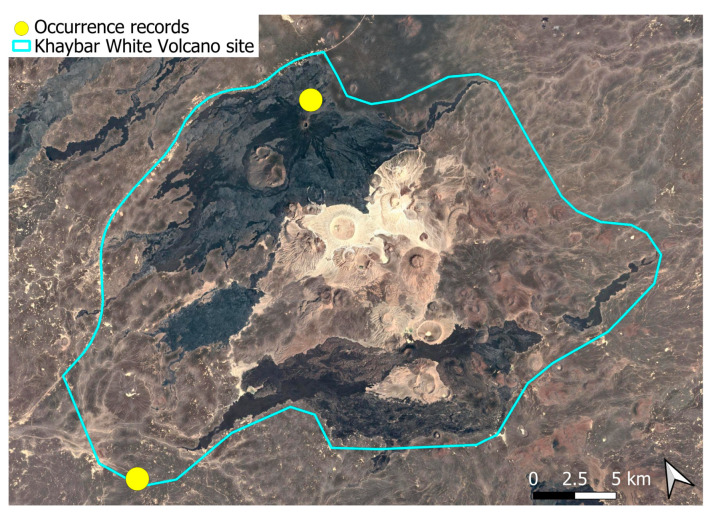
Distribution of *Syntrichia pagorum* (Milde) J.J. Amann in Khaybar White Volcano Geopark.

A subcosmopolitan taxon traditionally reported as corticolous, but observed here in a saxicolous context. In the Khaybar White Volcano Geopark (Figure 39), the species was very rare, occurred in very small quantities, and was consistently sterile.

From a taxonomic perspective, it remains a controversial entity. However, following Kürschner & Frey [5], it is here treated as a distinct species, particularly as it occurs independently from *Syntrichia laevipila*, with which it is often confused. The constant presence of gemmae supports its recognition as a separate taxon. Nonetheless, this is a morphologically complex group, and its taxonomy within the Arabian Peninsula remains in need of thorough revision [36].

Ecologically, the species is primarily terricolous, typically occupying sheltered soil pockets beneath boulders, in lava flows, or near cave entrances.

### 2.39. Syntrichia rigescens (Broth. & Geh.) Ochyra (Pottiaceae)

**Figure 40:**
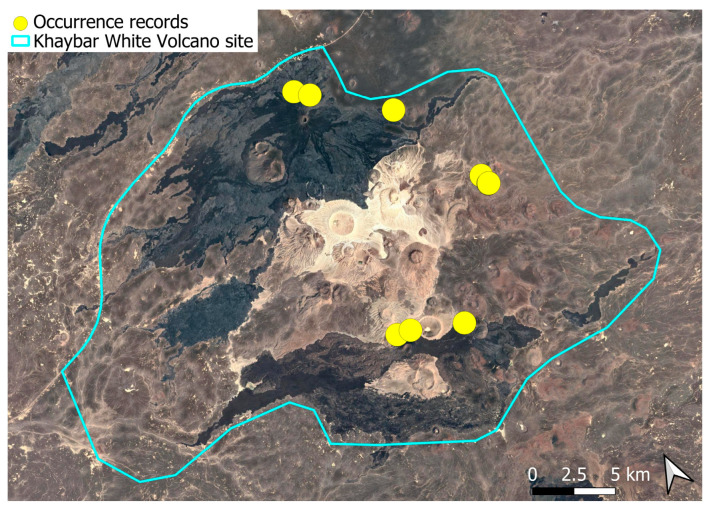
Distribution of *Syntrichia rigescens* (Broth. & Geh.) Ochyra in Khaybar White Volcano Geopark.

*Syntrichia rigescens* is a circum-Tethyan species previously recorded in Israel, Jordan, and Syria [5,37], and recently reported for the first time on the Arabian Peninsula in Hugonnot et al. [6]. It was rare and localized in the Khaybar White Volcano Geopark (Figure 40). Colonies were small and scattered, rarely covering more than 100 cm^2^ in a single patch, and were typically found in isolated occurrences. All observed individuals in the area were sterile.

Nevertheless, identification remains possible based on vegetative features [38,39]. Determination can be particularly challenging in the absence of gemmae, especially with respect to *S. handelii*, a species not yet recorded in the Arabian Peninsula. However, in *S. rigescens*, gemmae are almost always present, though often restricted to older leaves or confined to a small portion of the foliage. A distinguishing feature is the more strongly hispid dorsal surface of the costa in *S. rigescens*.

Ecologically, this species ranges from saxicolous to sub-terricolous, occupying exposed rocky substrates such as consolidated pozzolanic deposits, fissures, and lava flows, as well as nearby mineral soils.

### 2.40. Targionia hypophylla L. (Targioniaceae)

**Figure 41:**
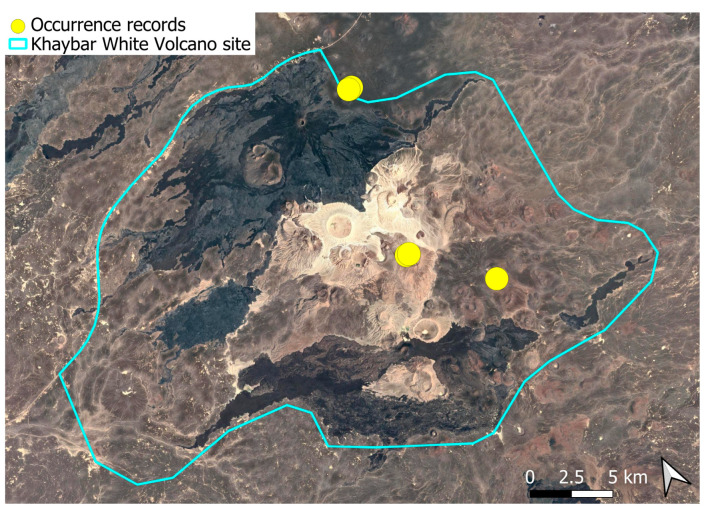
Distribution of *Targionia hypophylla* L. in and just outside Khaybar White Volcano Geopark.

*T. hypophylla* is a xerothermic Pangean taxon widespread on the Arabian Peninsula [3], though rare in and just outside Khaybar White Volcano Geopark (Figure 41). Populations were generally extremely sparse. Sporophyte production was occasional.

The genus *Targionia* has been the focus of detailed taxonomic studies on the Arabian Peninsula [40,41], leading to the description of the endemic subspecies *Targionia hypophylla* subsp. *linealis*. Despite targeted fieldwork in the Khaybar White Volcano Geopark, this subspecies was not observed. As emphasized by Hugonnot et al. [6], a comprehensive taxonomic reassessment of *Targionia* is warranted, particularly through the application of molecular tools to resolve existing ambiguities.

Ecologically, this liverwort is strictly saxicolous, occurring on volcanic slopes, exposed outcrops, and within fumarolic zones.

### 2.41. Timmiella barbuloides (Brid.) Mönk. (Pottiaceae)

**Figure 42:**
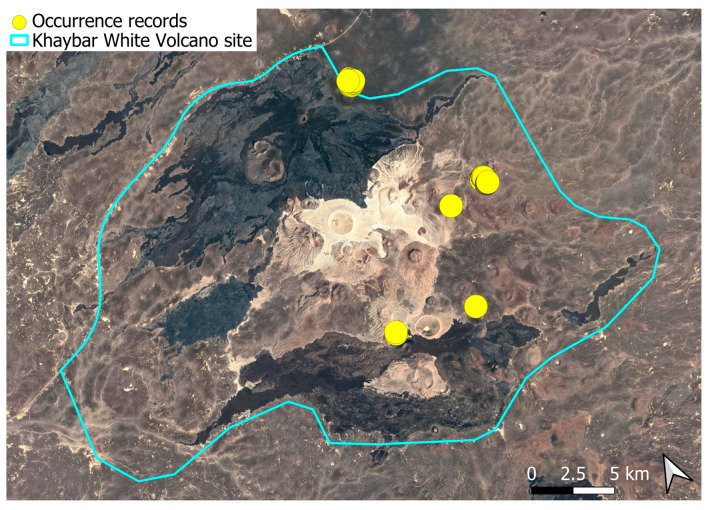
Distribution of *Timmiella barbuloides* (Brid.) Mönk. in and just outside Khaybar White Volcano Geopark.

This circum-Tethyan species is among the few bryophytes widely distributed across the Arabian Peninsula, particularly in the western Hijaz, Asir [3], and western AlUla County. In and just outside the Khaybar White Volcano Geopark, it was frequent and even locally abundant (Figure 42). Sporophytes were extremely rare, consistent with observations in AlUla [6].

In the few fertile collections, the species was confirmed to be paroicous, while all sterile material was likewise confidently assigned to the same taxon based on stable vegetative features. The only other representative of the genus in the Peninsula is *T. anomala* (Bruch & Schimp.) Limpr., known only in Kuwait [5].

Ecologically, the species is predominantly saxicolous, most often found on rock fissures, canyon walls, and mineral-rich surfaces. It also colonizes earthy microsites beneath boulders in lava fields and fumaroles.

### 2.42. Tortella nitida (Lindb.) Broth. (Pottiaceae)

**Figure 43:**
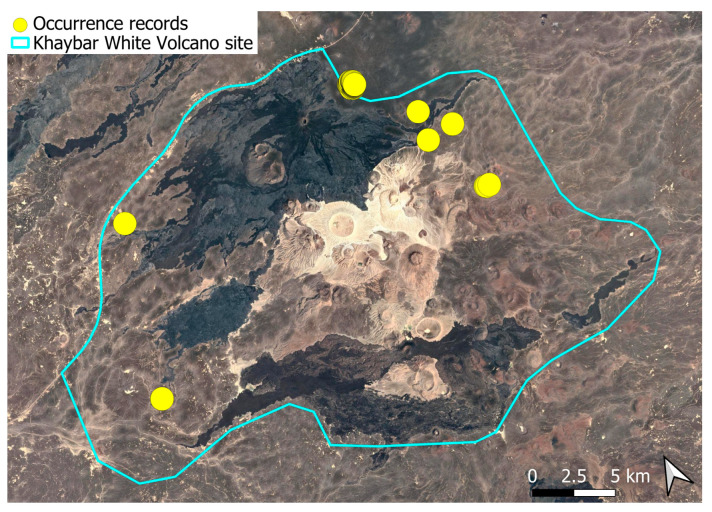
Distribution of *Tortella nitida* (Lindb.) Broth. in and just outside Khaybar White Volcano Geopark.

A circum-Tethyan species, this moss is rare across the Arabian Peninsula, with scattered records in the southwestern Asir Mountains (including Yemen), Socotra, and the United Arab Emirates [5]. In and just outside the Khaybar White Volcano Geopark (Figure 43), it was very frequent but spatially restricted. It occurred only in small tufts covering just a few square centimeters. It was consistently sterile, with no sporophytes observed.

Ecologically, the species is strictly saxicolous and displays a very narrow ecological amplitude. It is almost confined to fumarolic substrates in volcanic slopes.

### 2.43. Tortula atrovirens (Sm.) Lindb.

**Figure 44:**
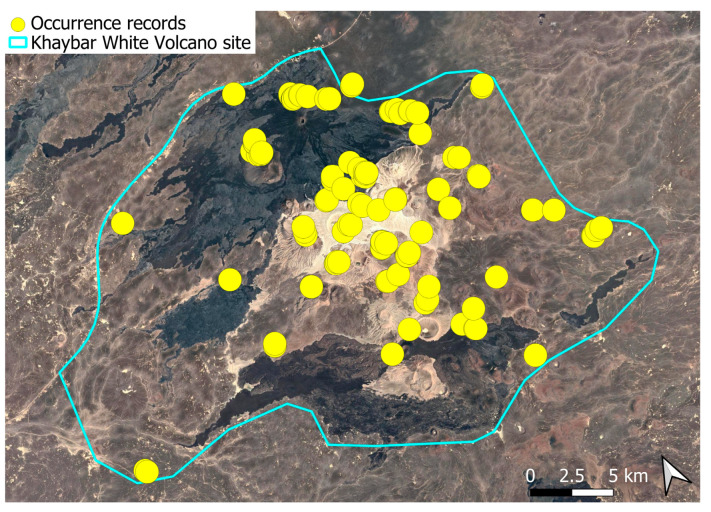
Distribution of *Tortula atrovirens* (Sm.) Lindb. in and just outside Khaybar White Volcano Geopark.

Five species of the genus *Tortula* have been recorded in this County. While members of this genus are more or less widespread across the Arabian Peninsula, they tend to be more abundant in mountainous regions such as the Asir, the Hijaz, and the cuesta of Jabal Tuwayq [3]. *T. atrovirens* is a xerothermic Pangean element. In and just outside the Khaybar White Volcano Geopark, *Tortula atrovirens* was of particular significance due to its frequency (Figure 44) and abundance. It produced sporophytes only occasionally.

Ecologically, it exhibits a broad amplitude, occurring on both earthy and rocky substrates. It is frequently found in rock fissures, canyon walls, and screes, but also in terricolous microhabitats such as earthy banks, flood-prone areas, and sheltered soil pockets beneath boulders.

### 2.44. Tortula inermis (Brid.) Mont. (Pottiaceae)

**Figure 45:**
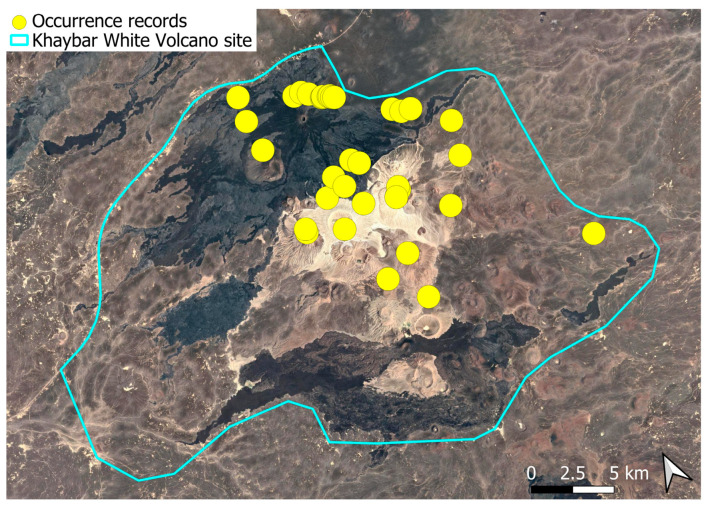
Distribution of *Tortula inermis* (Brid.) Mont. in Khaybar White Volcano Geopark.

A circum-Tethyan species, this moss is widely distributed across the Arabian Peninsula, with records from Oman and various regions of Saudi Arabia [3]. It was frequent and occurred mainly in the northeastern and central parts of Khaybar White Volcano Geopark (Figure 45). Most populations consisted of only a few scattered individuals. It was occasionally fertile, with sporophytes observed sporadically.

Ecologically, the species is primarily saxicolous, favoring exposed rocky substrates such as block faces, screes, and fissures. It also extends into nearby earthy microhabitats, including soil patches and wadi beds.

### 2.45. Tortula lindbergii Kindb. ex Broth. (Pottiaceae)

**Figure 46:**
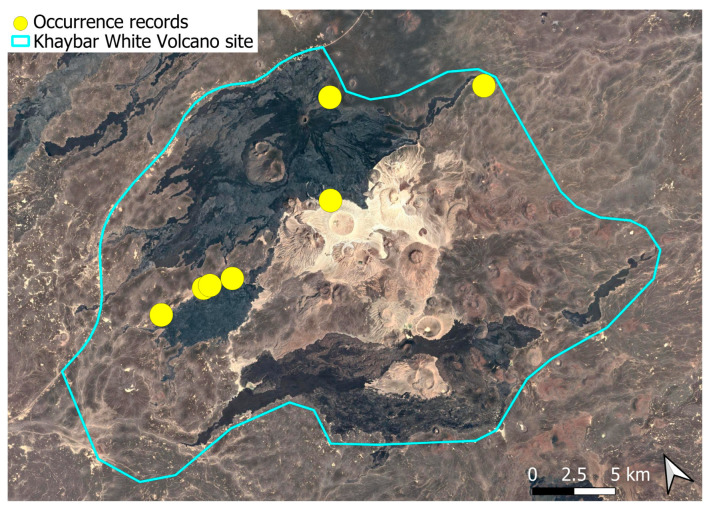
Distribution of *Tortula lindbergii* Kindb. ex Broth. in Khaybar White Volcano Geopark.

A circum-Tethyan taxon with a northern character, this species is known in Kuwait and the UAE [5], but remains rare and localized across the Arabian Peninsula. It was spatially localized and rare in Khaybar White Volcano Geopark (Figure 46). Populations were typically well defined and locally abundant, often forming pure stands covering several square meters. Individuals within these patches were usually numerous and robust. It was often fertile, though sporophyte development frequently aborted before maturation.

The well-developed peristome is a key diagnostic feature and proves invaluable for reliable identification.

Ecologically, this moss is distinctly terricolous, mainly growing on soil patches in flood-prone lava fields, but also on screes, and sheltered earthy microsites beneath boulders.

### 2.46. Tortula mucronifera W.Frey, Kürschner & Ros (Pottiaceae)

**Figure 47:**
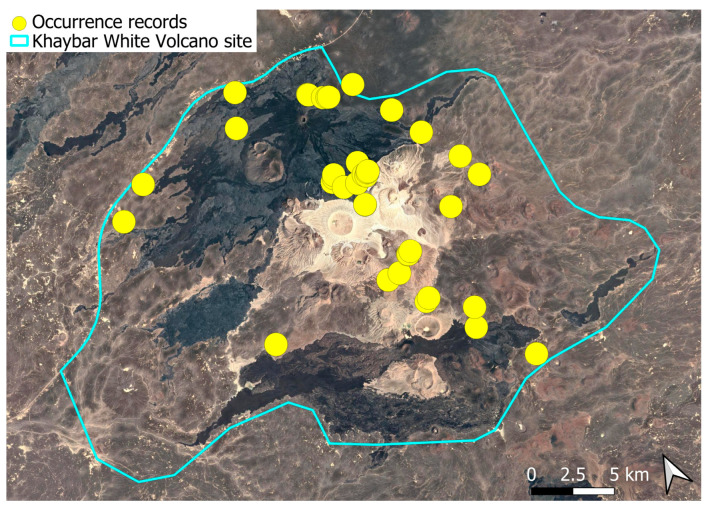
Distribution of *Tortula mucronifera* W.Frey, Kürschner & Ros in and just outside Khaybar White Volcano Geopark.

An endemic species to the Arabian Peninsula and Jordan, this moss is relatively widespread across the Peninsula [3,42]. It was frequent in and just outside Khaybar White Volcano Geopark (Figure 47). Populations remained consistently small, typically composed of only a few individuals. Sporophytes were frequently observed.

Ecologically, the species is terricolous to sub-saxicolous, most commonly found on earthy patches, at the base of rock faces, or beneath stones—particularly in wadis and among lava flows.

### 2.47. Tortula muralis Hedw. var. muralis (Pottiaceae)

**Figure 48:**
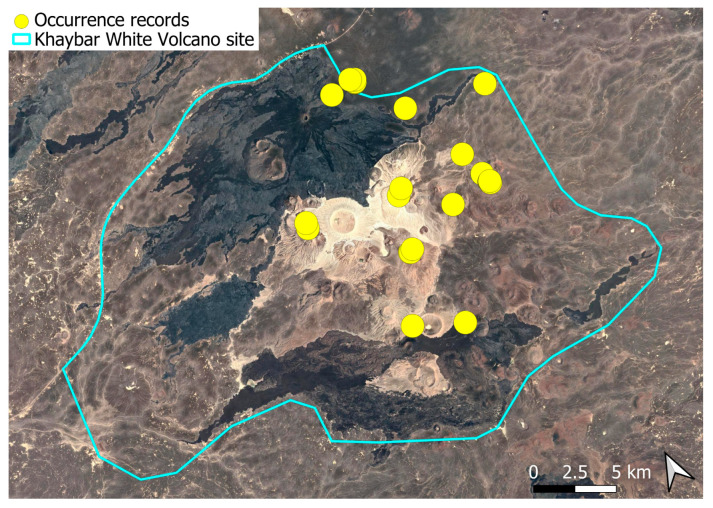
Distribution of *Tortula muralis* Hedw. var. *muralis* in and just outside Khaybar White Volcano Geopark.

A cosmopolitan taxon, this species appears to be rare in the Arabian Peninsula, with confirmed records only in Saudi Arabia—curiously absent from both Oman and Yemen [5]. It was moderately frequent and of restricted occurrence in and just outside Khaybar White Volcano Geopark (Figure 48). It was generally present as isolated tufts and was frequently observed with sporophytes.

In the area, it was stenotypic, occurring as a miniature expression, possibly reflecting adaptation to the region’s extreme aridity.

Ecologically, the species is highly versatile, colonizing a broad array of substrates including rock block faces, earthy banks, and soil patches in grasslands and wadis. It occupies both fully exposed lithic surfaces and more protected terricolous microsites.

### 2.48. Trichostomopsis australasiae (Hook. & Grev.) H. Rob. (Pottiaceae)

**Figure 49:**
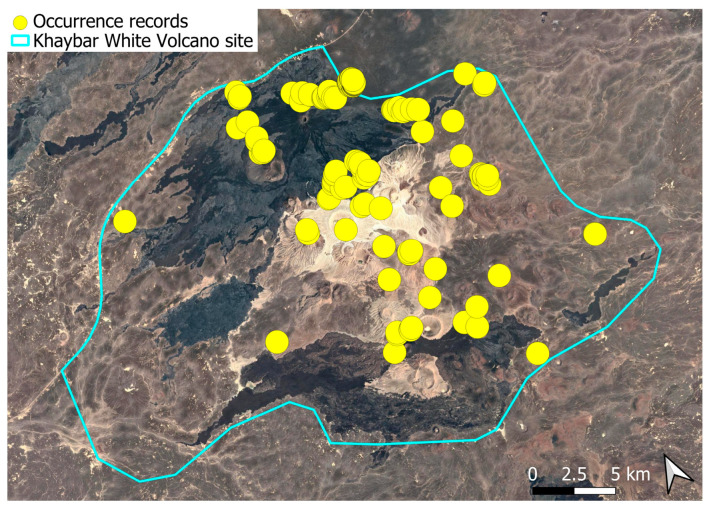
Distribution of *Trichostomopsis australasiae* (Hook. & Grev.) H. Rob. in and just outside Khaybar White Volcano Geopark.

This is one of the most common bryophyte species in AlUla County and across the Arabian Peninsula [3]. Like many xerophytic taxa, it is most frequently encountered in Jabal Tuwayq and the Asir Mountains. It belongs to a morphologically complex genus currently interpreted as containing only two species in the region, the other being *T. umbrosa* (Müll. Hal.) H. Robins., which is far more restricted in distribution and known only in Saudi Arabia. This xerothermic Pangean taxon was very frequent and widespread in and just outside Khaybar White Volcano Geopark (Figure 49). It was generally an abundant species, making relatively large populations (several tenths of cm^2^). Locally, the species was never observed with sporophytes, and sporophyte production appears to be rare on a global scale.

*T. australasiae* is a polymorphic species. Differentiation from *T. umbrosa* is challenging under field and even microscopic conditions: the hyaloderm is often difficult to observe, papillosity is weak or absent, and the basal marginal border—generally used as a taxonomic character—is inconsistently expressed. In the best-developed leaves, a partially differentiated, sometimes discoloured border may be present, whereas in smaller leaves it is entirely absent.

Ecologically, *T. australasiae* is predominantly saxicolous, thriving on rocky outcrops, fissures, and consolidated volcanic surfaces. It also extends into adjacent earthy microhabitats beneath boulders and within screes, especially in canyons and lava fields.

### 2.49. Tuerckheimia svihlae (E.B. Bartram) R.H. Zander (Pottiaceae)

**Figure 50:**
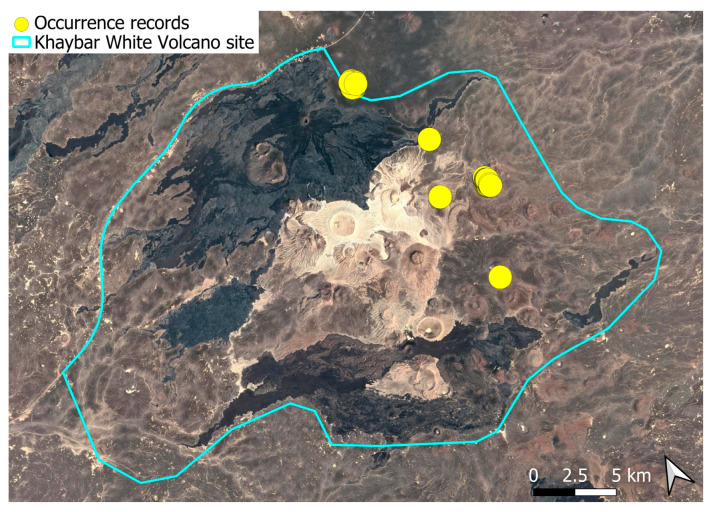
Distribution of *Tuerckheimia svihlae* (E.B. Bartram) R.H. Zander in and just outside Khaybar White Volcano Geopark.

This taxon, an American and Asian tropical species, is not known in Africa and is extremely localized on the Arabian Peninsula, with a single documented occurrence in Yemen [43]. In and just outside Khaybar White Volcano Geopark, it was very frequent and was observed to be sterile, but it often formed extensive colonies, covering all suitable microhabitats within its limited range (Figure 50). It was consistently sterile locally.

Ecologically, the species is strictly saxicolous, confined to fumaroles in volcanic slopes.

### 2.50. Vinealobryum vineale (Brid.) R. H. Zander (Pottiaceae)

**Figure 51:**
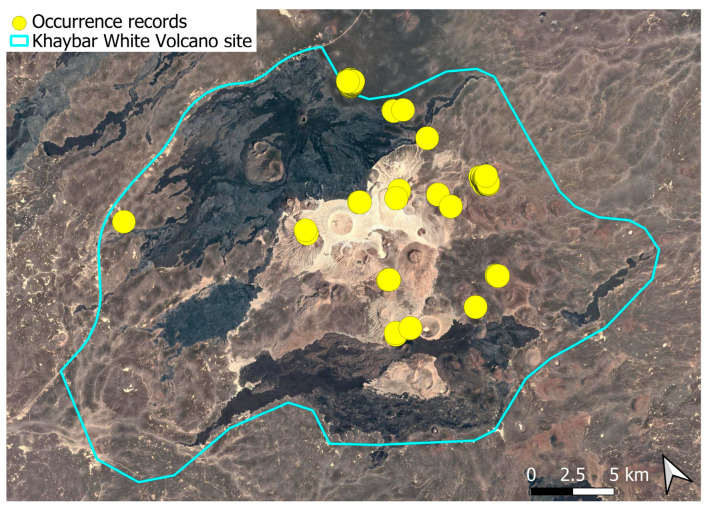
Distribution of *Vinealobryum vineale* (Brid.) R. H. Zander in and just outside Khaybar White Volcano Geopark.

This circum-Tethyan species has a scattered distribution across the Arabian Peninsula [3], as is the case in and just outside Khaybar White Volcano Geopark, where it was very frequent (Figure 51). There, it occurred in small, clustered populations that were consistently sterile.

It is readily distinguishable in mixed Pottiaceae communities due to the characteristic rusty coloration of its leaves, which provides a helpful visual cue in the field. Identification is further supported by the constant presence of a small translucent apical window composed of smooth cells within the leaf canal. Taxonomically, it is reliably separated from the closely related *Vinealobryum insulanum* (De Not.) R. H. Zander by the quadrate dorsal costal cells, whereas *V. insulanum* has elongated dorsal cells.

Ecologically, this species prefers dry, mineral-rich substrates and occurs in both terricolous and saxicolous microhabitats. It is frequently found on rocky outcrops, block faces, and soil beneath boulders, particularly in arid zones such as wadis and lava flows, but also in fumaroles.

### 2.51. Weissia condensa (Voit) Lindb. (Pottiaceae)

**Figure 52:**
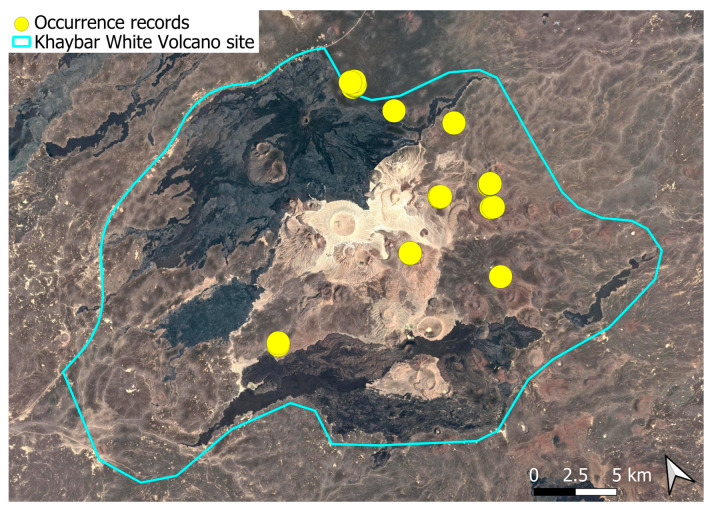
Distribution of *Weissia condensa* (Voit) Lindb. in and just outside Khaybar White Volcano Geopark.

This circum-Tethyan species is relatively common in the mountainous regions of the Arabian Peninsula [3] and occurred moderately frequently in Khaybar White Volcano site (Figure 52). It formed small tufts several cm^2^ wide. In and just outside the Khaybar White Volcano Geopark, the species was rarely observed with sporophytes.

It belongs to a huge and taxonomically challenging genus, which would benefit from a thorough revision at the scale of the Arabian Peninsula or more broadly. The local specimens were attributed to this species primarily based on vegetative features—most notably the presence of relatively short leaves with an obtuse apex and a distinctly wide costa (exceeding 70 µm at the base). Previous reports of *Weissia latiuscula* Müll. Hal. from the Peninsula have been shown to be erroneous, as the specimens referred to this name actually represent short-leaved forms of *W. condensa* [5].

Ecologically, *W. condensa* is primarily saxicolous, colonizing rocky outcrops, volcanic slopes, and fissured cliffs. It also occurs in more sheltered microhabitats such as soil beneath boulders.

## 3. Discussion

**Figure 53:**
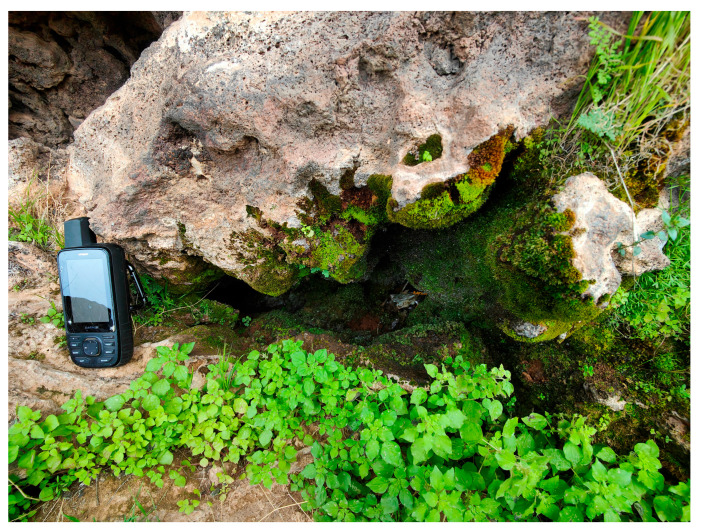
Fumarole in the Grey Volcano of the Khaybar White Volcano site.

The spatial patterns observed in the Khaybar White Volcano Geopark revealed a pronounced concentration of bryophyte richness, including tropical and high heritage value taxa, in geomorphologically complex sectors characterized by active geothermal features. The northeastern and central-eastern parts of the site, which hosted the highest species counts, were also the most structurally heterogeneous areas, marked by elevated topography, rugged volcanic formations, and the presence of active fumaroles (Figure 53). These conditions appeared to provide a combination of microhabitat diversity, persistent humidity, and thermal buffering—key factors that likely supported both high overall species richness and the occurrence of ecologically sensitive or thermophilous species.

In contrast, peripheral grid cells—especially those situated in the southern and southwestern margins—were topographically flatter, dominated by old extensive, and eroded basaltic lava fields (harrats), and largely devoid of remarkable geomorphological features. These areas frequently lacked bryophyte records altogether or supported only impoverished assemblages, and consistently showed the absence of tropical and high heritage value taxa.

The comparative analysis of bryophyte assemblages from AlUla and Khaybar is equally particularly relevant in the Saudi Arabian context, where bryophyte diversity remains poorly documented and unevenly explored. AlUla was the subject of a previous detailed study [6]. AlUla and Khaybar represent two contrasting ecological systems within the same biogeographical region—one shaped by lithological heterogeneity, the other by volcanic and geothermal activity. A further point of contrast between the two sites lies in their spatial extent: while the AlUla study area covers approximately 22,561 km^2^, the Khaybar White Volcano Geopark occupies a much smaller surface of about 600 km^2^.

The dataset revealed both a comparable species richness (48 species at AlUla vs. 51 at Khaybar) and a marked ecological divergence between the two sites. Approximately one-third of the taxa (17 species) were shared, while each site also hosted a set of exclusive species reflecting distinct environmental conditions. The flora of AlUla was predominantly composed of pioneer terricolous species such as *Acaulon triquetrum*, *Encalypta vulgaris*, and *Entosthodon duriaei*, with additional taxa found on rock shelters or tufaceous substrates (*Clevea spathysii*, *Eucladium verticillatum*). These habitats, while ecologically diverse, were primarily linked to lithological features and typical of arid rocky environments.

The tropical species present at AlUla were mostly associated with temporarily moist habitats, primarily tufaceous substrates located in the deeply incised minor beds of wadis in the far western part of the study area (Hijaz range). These mountainous sectors are subject to moderate monsoonal influences. Their ecological context is entirely unrelated to geothermal activity, and bears no resemblance to fumarolic habitats.

In contrast, the Geopark site was floristically distinguished by the presence of species strictly restricted to fumaroles. These geothermal microhabitats, characterized by elevated humidity and ground temperatures, created refugial conditions that allowed the persistence of tropical and thermophilous bryophytes. All tropical taxa observed at Khaybar (*Anoectangium eruchloron*, *Fissidens sciophyllus*, *Plagiochasma eximium*, etc.) were found exclusively in these active fumarolic zones, which are entirely absent from AlUla or from the Arabian Peninsula. This singular ecological setting explains the occurrence of species otherwise unknown in the region and supports Khaybar’s status as a center of floristic originality and national importance. These findings highlighted the disproportionately high bryophyte diversity of Khaybar White Volcano Geopark relative to its limited area, emphasizing the key role of geothermal microhabitats in shaping species richness.

As already noted for the AlUla region [6], several taxa—including *Didymodon* cf. *rigidulus*, *Entosthodon* cf. *commutatus*, and *Fissidens* cf. *arnoldii*—present identification challenges due to morphological ambiguity. In addition, particular species that appear widespread or poorly defined, such as *Fissidens sciophyllus*, raise questions about their taxonomic delimitation. Resolving these uncertainties will require integrative approaches, particularly molecular analyses, as traditional morphology-based identification proves insufficient in these morphologically reduced organisms [44,45]. These issues will be addressed in detail in future studies.

To date, no conservation actions specifically targeting bryophytes have been implemented in Saudi Arabia, including in the Khaybar White Volcano Geopark. However, the conservation of bryophytes in hyper-arid environments is crucial. These organisms, despite their small size and inconspicuous appearance, play a fundamental ecological role in soil stabilization, water retention, erosion control, and microhabitat creation for microorganisms and invertebrates [46,47,48].

Bryophyte richness, including tropical and high-heritage value taxa, was concentrated in topographically elevated and geomorphologically complex areas with volcanic features and geothermal activity. The northeastern and central-eastern sectors, which showed the highest species diversity, aligned with these structurally heterogeneous zones. In contrast, the flatter southern and southwestern margins, dominated by basaltic lava fields, were species-poor and lacked notable taxa. Unlike other parts of the Arabian Peninsula, such as AlUla, where tufaceous deposits in shaded wadi beds support temporary moisture-dependent assemblages, the Geopark lacks significant tufa formations and features an extremely sparse hydrographic network. This makes its bryophyte communities especially vulnerable to grazing pressure and mechanical disturbances.

Overgrazing by camels, goats, and donkeys severely affected both terricolous and saxicolous species by trampling, compacting substrates, and destabilizing the microstructures essential for bryophyte establishment and spore germination [48,49]. Fissures between lava blocks, lightly stabilized soil patches, and fumarole margins represent critical microhabitats that can be irreversibly degraded by livestock movement, off-road vehicle traffic, and unregulated visitor access.

Moreover, volcanic landscapes such as Khaybar are increasingly impacted by anthropogenic pressures, including stone rearrangement, trail erosion by hikers, camping activities, and the use of vehicles in lava flows. These activities threaten the fragile spatial configuration of habitats essential for bryophyte persistence. While pioneer species may temporarily benefit from light surface disturbance, sustained pressure leads to diversity loss and local extinctions. The situation is further exacerbated by climate change, as bryophytes—being poikilohydric—are highly sensitive to shifts in temperature and humidity [47,50]. Frequent observations of abortive sporophytes (e.g., in *Entosthodon*, *Bryum*, and various *Pottiaceae*) indicate that many species fail to complete their reproductive cycles under current conditions.

The observed strong spatial correlation between overall species richness and the number of tropical and high heritage value taxa suggests that floristic richness at the grid cell scale is a robust proxy for conservation value. This insight provides a valuable tool for identifying priority zones for protection. In this respect, the northeastern quadrant of the Khaybar site emerges as a key conservation area, combining high bryophyte richness, ecological originality, and the presence of geographically restricted species.

In addition, the markedly depauperate bryophyte flora of marginal zones may also reflect historical land use impacts. These areas have likely been subject to prolonged grazing pressure, leading to the degradation of soil properties and the loss of vascular plant cover that could otherwise offer protective microhabitats. Such degradation is particularly detrimental to bryophytes, which are highly sensitive to microclimatic fluctuations and physical disturbance.

Given the uniqueness and vulnerability of the Khaybar bryoflora, long-term monitoring is urgently required, particularly in fumarolic zones where microhabitats are most fragile. Conservation measures should combine habitat protection, regulated access, and the integration of bryophytes into broader biodiversity management frameworks. Priority actions include fencing the fumarole fields of the northeastern quadrant to exclude grazing livestock and establishing designated visitor pathways to prevent trampling of soil crusts and fumarolic margins. The records obtained from this study will serve as a basis for identifying key conservation areas within the Geopark as its regulations and management guidelines are developed.

**Figure 54:**
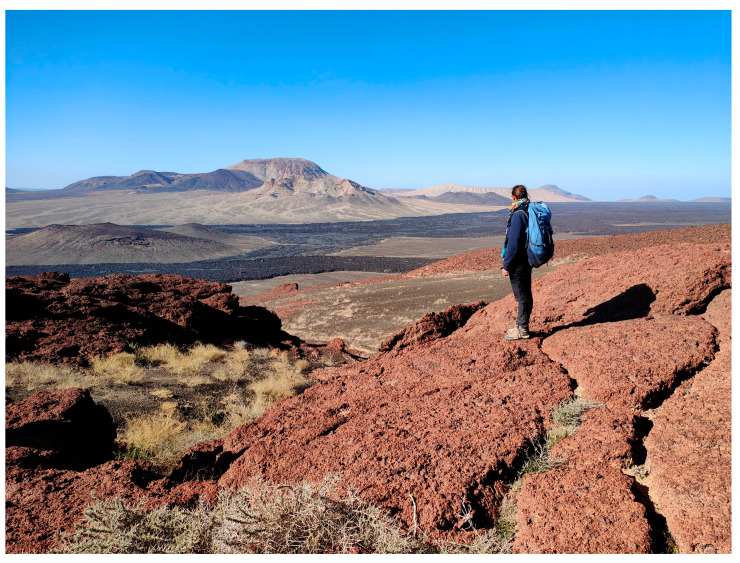
Volcanic landscape of the Khaybar White Volcano Geopark.

Building on the recently published checklist of Saudi Arabia’s bryophytes [6], we provide an updated inventory for the Khaybar White Volcano site here. A total of 51 bryophyte species were recorded, comprising five liverworts and 46 mosses. This diversity represents approximately 30% of the country’s known bryophyte flora, concentrated within an area covering less than 0.3% of the national territory. Notably, two of the twelve bryophyte taxa currently considered endemic to the Arabian Peninsula (*Crossidium deserti* and *Tortula mucronifera*) have been recorded at Khaybar. The site’s floristic importance is further underscored by the discovery of 12 species newly recorded for Saudi Arabia, including six that are new to the entire Arabian Peninsula. These findings not only highlight the unexpected richness of bryophytes in a hyper-arid context such as Khaybar, but also emphasize the urgent need for a systematic, large-scale recording effort—ideally at the Arabian Peninsula level—to adequately document the region’s underexplored bryophyte diversity (Figure 54).

## 4. Materials and Methods

### 4.1. Study Site

**Figure 55:**
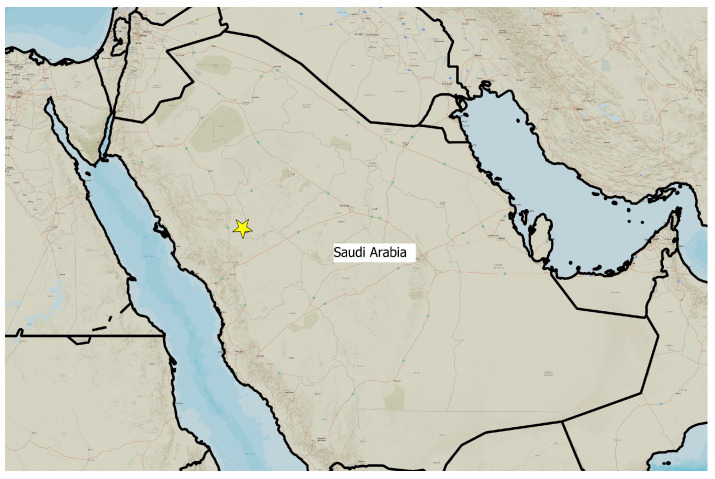
Location of Khaybar White Volcano Geopark within the Arabian Peninsula (indicated by a yellow star).

Khaybar White Volcano Geopark, situated in northwestern Saudi Arabia (Figure 55) within the Saharo–Sindian biogeographic zone [29,51], exhibits a hyper-arid climate. Total annual precipitation is approximately 70–100 mm, with most rainfall occurring in winter months (e.g., January–February) and negligible precipitation through spring to fall. Interannual variability is pronounced, featuring prolonged dry spells and sporadic wet events, characteristic of this bioclimatic region.

Harrat Khaybar is one of the largest lava fields in Saudi Arabia, covering around 20,564 km^2^, and formed roughly 10 million years ago until recent times [9,52]. The lava fields include many different types of volcanoes, along with extensive lava flows, both old and recent. The volcanic landscape resulted from different phases of eruptions, the first around 10 million years ago [52], a second around 5 to 3 million years ago, another phase of eruptions 3 to 1 million years ago, and the latest phase of mafic volcanoes from around 1 million years ago to recent [53]. This large-scale volcanic activity resulted from the rifting of the Red Sea, which caused faulting and melting of the continental crust [10]. Within this large harrat, the Royal Commission for AlUla manages a 600 km^2^ area, known as Khaybar White Volcano Geopark (Figure 55). The Geopark contains basalt volcanoes and lava flows in proximity to silicic-rich comendite volcanoes from the remelting of Precambrian basement rocks [54], creating a dramatically contrasting landscape. The area includes a wide range of geodiversity for a relatively small area, with different monogenetic and polygenetic volcano types, including calderas, splatter cones, scoria cones, lava domes, shield volcanoes, and pyroducts (lava tubes) [9]. Many combined factors have created a wide variety of different habitats within Khaybar White Volcano Geopark. Older, eroded volcanoes create more valleys, crevices, and loose material than younger volcanoes. The different volcano types additionally erode differently, creating different habitats ranging from blocky boulders to crevices and scree material. A predominantly arid environment results in wadis due to flash flooding, and fine wind blow sediment settling in pockets on younger lava flows: an example of two drastically different, distinct habitats.

The climate across most of northern Arabia features very hot, dry summers and cool to warm winters. Precipitation mainly comes from cyclonic depressions during winter and spring, originating in the Atlantic or Mediterranean. Rainfall is irregular and unpredictable in timing, quantity, and distribution, often falling as heavy, localized showers. Some areas may receive almost no rain in certain years.

**Figure 56:**
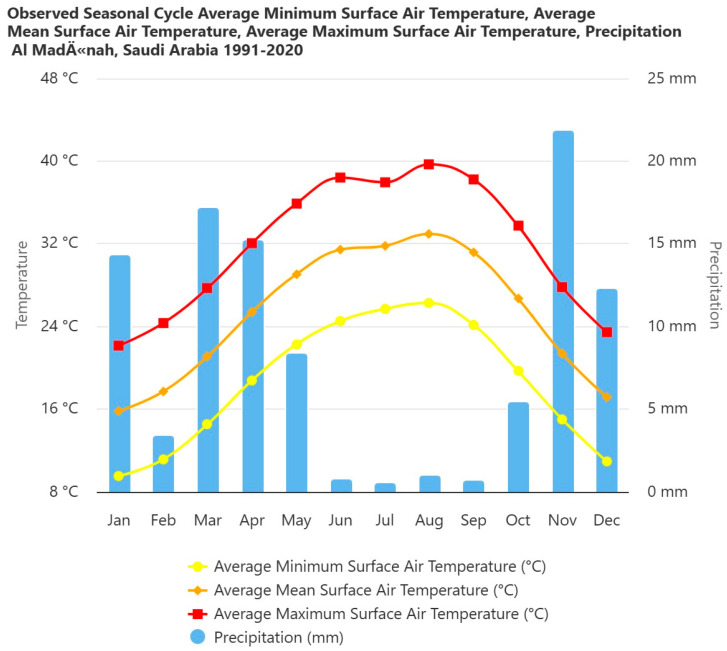
Climatogram for Al Madinah (Data: 1991–2021; Climate Change Knowledge Portal: Observed Climate Data, ERA5 0.25-Degree, https://doi.org/10.57966/128g-6s70 (accessed on 12 July 2025).

Meteorological data from the Al Madinah station provide a good representation of the regional climate (Figure 56), with a mean annual rainfall of 94 mm and a mean annual temperature of 27 °C.

In Khaybar White Volcano site, the 5 main types of tracheophyte vegetation units are the following [55]:

- Community 1: desert community with *Artemisia judaica*, belonging to the Middle Eastern artemisian steppes, typical of sandy areas and semi-arid piedmonts (typical species: *Achillea fragrantissima*–*Artemisia judaica*–*Pulicaria undulata*);
- Community 2: desert shrub community belonging to the xerophilous Saharo-Sindian formations, typical of rocky piedmonts and stony depressions (typical species: *Ochradenus baccatus*–*Lycium shawii*–*Zilla spinosa*);
- Community 3: desert herb–shrub community belonging to the Saharo-Sindian psammophilous–gypsicolous formations, typical of sandy and gypseous plains (typical species: *Ducrosia flabellifolia*–*Scrophularia deserti*–*Gypsophila capillaris*);
- Community 4: nitrophilous desert ruderal community belonging to the Saharo-Sindian pioneer formations on disturbed and nutrient-enriched soils, typical of sandy depressions and field margins (typical species: *Malva parviflora*–*Farsetia aegyptia*);
- Community 5: open desert woodland community belonging to the Saharo-Sindian formations of wadis and gravelly plains (typical species: *Acacia tortilis*–*Peganum harmala*–*Zygophyllum bruguieri*).

**Figure 57:**
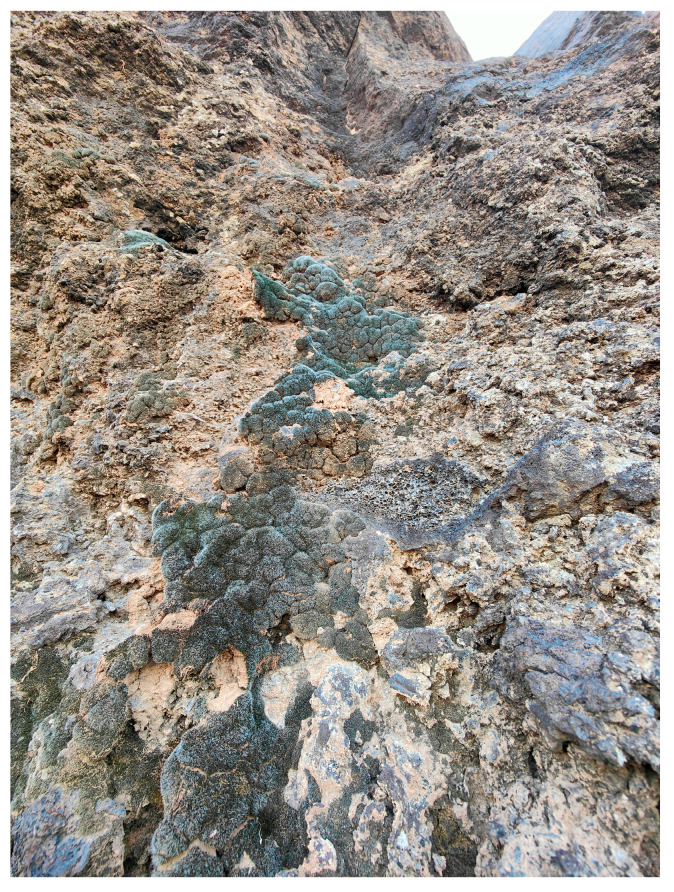
*Grimmia orbicularis* rock-dwelling community in a deeply incised temporary wadi.

It must be acknowledged, however, that many bryological communities develop in the absence of any associated tracheophytic cover (Figure 57), and therefore cannot be assigned to any previously described vegetation type.

### 4.2. Surveys and Nomenclature

**Figure 58:**
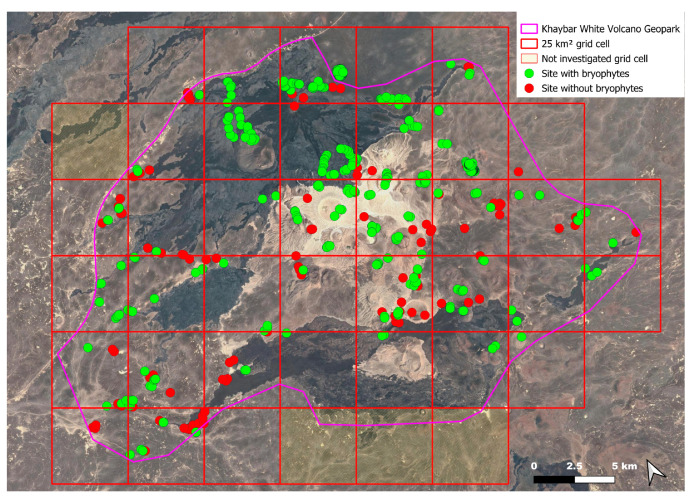
Location of surveyed sites.

The surveys were conducted from 21–23 February 2024, and 15 February to 17 March 2025, with 551 selected sites surveyed (Figure 58). To ensure a systematic and comprehensive bryofloristic inventory of the study area, a regular grid composed of 5 × 5 km cells was superimposed on high-resolution aerial imagery. This grid provided a spatial framework to structure the field campaign, enabling homogeneous coverage of the entire site and facilitating logistical planning. Whilst the primary focus was the Khaybar White Volcano Geopark area, the boundary of which can be seen in Figure 58, the 5 × 5 km cells did extend outside the Geopark area. These areas were surveyed with the same diligence as those inside the Geopark boundary.

Preliminary analysis of the aerial photographs allowed the identification of landscape features likely to support bryophyte diversity, including north-facing rocky slopes, coarse block screes, wadi networks, volcanic structures, and zones characterized by unusual geological substrates or enhanced moisture retention. Accessibility via existing 4 × 4 tracks was also evaluated beforehand to optimize travel routes and improve field efficiency. Information from local rangers about safe access for prospecting and the presence of potential habitats for bryophytes was included in the site selection.

Within each 25 km^2^ grid cell, a standardized methodology was applied, combining vehicular and pedestrian exploration, targeted inspection of pre-identified microhabitats, and opportunistic prospection of favorable sites. In situ bryosociological relevés were carried out [56] using adapted abundance–dominance coefficients [57]. Concurrently, key ecological parameters were recorded (e.g., slope, exposure, vegetation cover, substrate type). Habitat (boulder scree; cave entrance; cliff complex; flood-prone lava flow; grassland; lava flow; rocky canyon; seasonally wet area; volcanic slope; wadi) and microhabitat (consolidated pozzolanic deposit; earthy banks; earthy patch; earthy patch beneath boulders; exposed mudflat; fumarole; rock block face; rock fissures; rock outcrop; soil shelter beneath a lava slab) types were recorded. The frequency of occurrence of each species was calculated for both habitat and microhabitat levels. This approach allowed us to assess patterns of bryophyte distribution across broader ecological units (habitats) as well as finer-scale environmental conditions (microhabitats). Frequencies were expressed as the proportion of relevés in which a given species was recorded within each defined category. Voucher specimens were collected for laboratory identification. Systematic photographic documentation was performed for both habitats and microhabitats.

Nearly all of the 41 grid cells were surveyed, except for four that were too remote to access. A standardized protocol was applied to ensure methodological consistency across the study area: all principal bryophyte-bearing microhabitats (rocks, soil crusts, shaded crevices, ephemeral streambeds, fumarolic deposits, etc.) were systematically examined, with proportionally greater effort devoted to the richest or most bryologically diverse habitats. Survey effort within each grid cell was continued until no new species were encountered across all major pre-identified microhabitat types, such as wadi beds, fumarolic slopes, and rocky outcrops, thereby ensuring a high degree of completeness despite intrinsic landscape heterogeneity. A total of 450 bryosociological relevés were systematically taken, which corresponds to 1806 floristic data.

The definition of the Arabian Peninsula follows [3] and, therefore, excludes Iraq and Jordan. The taxonomy and nomenclature of the bryophytes mainly follow [5], except for the 13 names indicated in Table 2, for which specific references are provided. For vascular plants, we use [58]. The vouchers are currently kept in the private herbarium of Vincent Hugonnot and Florine Pépin and will be transferred to the Royal Commission for AlUla’s (RCU) collection in early 2026.

Several species are taxonomically problematic and are currently under reassessment. Such taxa have been named using up-to-date treatments, i.e., [5], including *Didymodon* cf *rigidulus* Hedw. *Fissidens* cf. *arnoldii* R. Ruthe and *Entosthodon* cf. *commutatus* Durieu & Mont. The latter is not mentioned in [5].

Each species is examined in terms of its biogeographical affinities, distribution on the Arabian Peninsula and in the Khaybar White Volcano site, frequency, fertility, morphological plasticity or polymorphism, and its taxonomy. The biogeographical elements are mostly extracted from [20]. The number of observations recorded for each species is provided as a measure of its frequency within the study area. Tropical, new for Saudi Arabia or new for Arabian Peninsula species are here considered as high heritage value species.

To assess the relative rarity of bryophyte species recorded, we classified species into five rarity classes based on the total number of observations. The number of observations for each species was log-transformed using the function log_10_(1 + x) to reduce the influence of highly dominant species and to reflect abundance gradients better. Rarity classes were then defined using quintile thresholds of the transformed values:

- R1—Very rare: bottom 20% of log-transformed frequencies;
- R2—Rare: 20–40%;
- R3—Moderately frequent: 40–60%;
- R4—Frequent: 60–80%;
- R5—Very frequent: top 20%.

Each species was mapped individually, with yellow dots indicating all recorded localities. In some cases, points may overlap due to the geographical proximity of the sites. The boundary of the Khaybar White Volcano Geopark is shown on every map.

## Figures and Tables

**Table 1:** Taxonomy and frequency of bryophyte taxa observed in Khaybar site (see Section 4, Materials and Methods).

Accepted Nomenclature	Number of Observations	Rarity (5 Classes)	Taxonomy	Family	Tropical Species	New for KSA	New for the Arabian Peninsula
*Aloina rigida*	12	R3—Moderately frequent	Moss	Pottiaceae			
*Anoectangium aestivum*	4	R2—Rare	Moss	Pottiaceae		+	
*Anoectangium euchloron*	52	R4—Frequent	Moss	Pottiaceae	+	+	+
*Bryum dichotomum*	162	R5—Very frequent	Moss	Bryaceae			
*Crossidium aberrans*	19	R3—Moderately frequent	Moss	Pottiaceae			
*Crossidium crassinervium*	53	R4—Frequent	Moss	Pottiaceae			
*Crossidium deserti*	8	R2—Rare	Moss	Pottiaceae			
*Crossidium squamiferum*	35	R4—Frequent	Moss	Pottiaceae			
*Didymodon cf rigidulus*	48	R4—Frequent	Moss	Pottiaceae			
*Didymodon desertorum*	35	R4—Frequent	Moss	Pottiaceae			
*Entosthodon* cf. *commutatus*	82	R5—Very frequent	Moss	Funariaceae			
*Entosthodon muhlenbergii*	10	R2—Rare	Moss	Funariaceae			
*Fissidens* cf. *arnoldii*	12	R3—Moderately frequent	Moss	Fissidentaceae			
*Fissidens sciophyllus*	24	R3—Moderately frequent	Moss	Fissidentaceae	+		
*Fissidens crispus*	3	R1—Very rare	Moss	Fissidentaceae			
*Fossombronia caespitiformis subsp. caespitiformis*	17	R3—Moderately frequent	Liverwort	Fossombroniaceae			
*Funaria hygrometrica*	3	R1—Very rare	Moss	Funariaceae			
*Geheebia erosa*	1	R1—Very rare	Moss	Pottiaceae		+	+
*Grimmia capillata*	1	R1—Very rare	Moss	Grimmiaceae		+	+
*Grimmia orbicularis*	114	R5—Very frequent	Moss	Grimmiaceae			
*Gymnostomum calcareum var. calcareum*	18	R3—Moderately frequent	Moss	Pottiaceae			
*Gymnostomum mosis*	48	R4—Frequent	Moss	Pottiaceae			
*Husnotiella revoluta*	12	R3—Moderately frequent	Moss	Pottiaceae	+	+	
*Hymenostylium hildebrandtii*	2	R1—Very rare	Moss	Pottiaceae	+		
*Molendoa sendtneriana*	3	R1—Very rare	Moss	Pottiaceae	+	+	+
*Microbryum davallianum*	2	R5—Very frequent	Moss	Pottiaceae			
*Microbryum starckeanum*	96	R1—Very rare	Moss	Pottiaceae			
*Plagiochasma eximium*	1	R1—Very rare	Liverwort	Aytoniaceae	+		
*Plagiochasma rupestre*	1	R1—Very rare	Liverwort	Aytoniaceae			
*Pterygoneurum subsessile*	10	R2—Rare	Moss	Pottiaceae		+	+
*Ptychostomum pseudotriquetrum*	5	R2—Rare	Moss	Bryaceae			
*Ptychostomum torquescens*	97	R5—Very frequent	Moss	Bryaceae		+	+
*Riccia cavernosa*	12	R3—Moderately frequent	Liverwort	Ricciaceae			
*Scorpiurium circinatum*	32	R4—Frequent	Moss	Brachytheciaceae			
*Syntrichia caninervis var. caninervis*	4	R2—Rare	Moss	Pottiaceae			
*Syntrichia fragilis*	1	R1—Very rare	Moss	Pottiaceae	+		
*Syntrichia laevipila*	4	R2—Rare	Moss	Pottiaceae			
*Syntrichia pagorum*	2	R1—Very rare	Moss	Pottiaceae		+	
*Syntrichia rigescens*	11	R2—Rare	Moss	Pottiaceae			
*Targionia hypophylla*	10	R2—Rare	Liverwort	Targioniaceae			
*Timmiella barbuloides*	50	R4—Frequent	Moss	Timmiellaceae			
*Tortella nitida*	70	R5—Very frequent	Moss	Pottiaceae		+	
*Tortula atrovirens*	102	R5—Very frequent	Moss	Pottiaceae			
*Tortula inermis*	38	R4—Frequent	Moss	Pottiaceae			
*Tortula lindbergii*	7	R2—Rare	Moss	Pottiaceae		+	
*Tortula mucronifera*	38	R4—Frequent	Moss	Pottiaceae			
*Tortula muralis var. muralis*	27	R3—Moderately frequent	Moss	Pottiaceae			
*Trichostomopsis australasiae*	130	R5—Very frequent	Moss	Pottiaceae			
*Tuerckheimia svihlae*	113	R5—Very frequent	Moss	Pottiaceae	+	+	
*Vinealobryum vineale*	135	R5—Very frequent	Moss	Pottiaceae			
*Weissia condensa*	29	R3—Moderately frequent	Moss	Pottiaceae			
51	51	51	51	51	8	12	6

**Table 2:** Nomenclature for names deviating from [5].

Current Nomenclature	Kürschner & Frey, 2020 [5]	Followed Reference
*Anoectangium euchloron* (Schwägr.) Spruce	not mentioned	[59]
*Didymodon desertorum* (J. Froehl.) J.A. Jiménez & M.J. Cano	not recognized	[60]
*Entosthodon commutatus* Durieu & Mont.	not mentioned	[61]
*Geheebia erosa* (J.A. Jiménez & J. Guerra) J.A. Jiménez & M.J. Cano	not mentioned	[25]
*Husnotiella revoluta* Cardot	*Didymodon revolutus* (Cardot) R.S. Williams	[25]
*Molendoa sendtneriana* (Bruch & Schimp.) Limpr.	not mentioned	[59]
*Ptychostomum pseudotriquetrum* (Hedw.) J.R. Spence & H.P. Ramsay	*Bryum pseudotriquetrum* (Hedw.) G. Gaertn.	[19]
*Ptychostomum torquescens* (Bruch & Schimp.) Ros & Mazimpaka	*Bryum torquescens* Bruch & Schimp.	[19]
*Syntrichia pagorum* (Milde) J.J. Amann	not recognized	[36]
*Trichostomopsis australasiae* (Hook. & Grev.) H. Rob.	*Didymodon australasiae* (Hook. & Grev.) R. H. Zander	[25]
*Vinealobryum vineale* (Brid.) R.H. Zander	*Didymodon vinealis* (Brid.) R. H. Zander	[25]

## Data Availability

The datasets presented in this article are not readily available because of the data ownership. Requests to access the datasets should be directed to the Vice President of Wildlife & Natural Heritage, RCU.
